# Inferring origin-destination distribution of agent transfer in a complex network using deep gated recurrent units

**DOI:** 10.1038/s41598-023-35417-9

**Published:** 2023-05-22

**Authors:** Vee-Liem Saw, Luca Vismara, Suryadi, Bo Yang, Mikael Johansson, Lock Yue Chew

**Affiliations:** 1 Division of Physics and Applied Physics, School of Physical and Mathematical Sciences, Nanyang Technological University, Singapore, Singapore; 2 School of Electrical Engineering, KTH Royal Institute of Technology, Stockholm, Sweden

**Keywords:** Mathematics and computing, Physics

## Abstract

Predicting the origin-destination (OD) probability distribution of agent transfer is an important problem for managing complex systems. However, prediction accuracy of associated statistical estimators suffer from underdetermination. While specific techniques have been proposed to overcome this deficiency, there still lacks a general approach. Here, we propose a deep neural network framework with gated recurrent units (DNNGRU) to address this gap. Our DNNGRU is *network-free*, as it is trained by supervised learning with time-series data on the volume of agents passing through edges. We use it to investigate how network topologies affect OD prediction accuracy, where performance enhancement is observed to depend on the degree of overlap between paths taken by different ODs. By comparing against methods that give exact results, we demonstrate the near-optimal performance of our DNNGRU, which we found to consistently outperform existing methods and alternative neural network architectures, under diverse data generation scenarios.

## Introduction

Deciphering the origin-destination (OD) pair has been at the heart of various protocols that aim to evaluate the traffic demand and flow within complex systems. Interest in OD pairs results from the basic information it encodes on the distribution of people, materials, or diseases which has direct bearing on the socioeconomic phenomena of human mobility, resource allocation, and epidemic spreading. An intrinsic utility in gaining knowledge of the OD distribution is that paths with higher transfer rates can be enhanced to improve system’s efficiency, or blocked to impede the transfer of malicious/undesirable entities. By far the most intensively studied OD problem for a complex system is the estimation of OD traffic of an Internet network from measurable traffic at router interfaces^1^. Collection of link traffic statistics at routers within a network is often a much simpler task than direct measurements of OD traffic. The collected statistics provide key inputs to any routing algorithm, via link weights of the open shortest path first (OSPF) routing protocol. Shortly after, similar techniques have been adapted for the problem of identifying transportation OD by measuring the number of vehicles on roads^2–5^. Such inference of vehicular OD has been used by Dey et al.^5^ to give better estimates of commuters’ travel time, or by Saberi et al.^6^ to understand the underlying dynamical processes in travel demand which evolve according to interactions and activities occurring within cities.

Several techniques have been developed in an effort to estimate OD information from link/edge counts. These include expectation-maximisation^7^, entropy maximisation (or information minimisation)^8–10^, Bayesian inference^3,11–13^, quasi-dynamic estimations^14,15^, and the gravity model^16–18^. As the number of OD degrees of freedom (quadratically proportional to number of nodes) generally outnumbers the number of link counts (linearly proportional to number of nodes), the major issue concerning OD estimation is that the problem is severely underdetermined. Vardi^7^ attempted to resolve this issue by treating the measurements of OD intensities as Poisson random variables, and used expectation-maximisation with moments to figure out the most likely OD intensities giving rise to the observed measurements of link counts. The non-unique solutions due to underdetermination had also been addressed through the principle of entropy maximisation, as well as by Bayesian inference through the assumption of prior OD matrices. Alternatively, ambiguities in OD inferences were treated by quasi-dynamic method using prior knowledge about historical trip data, or through parameters calibration of the gravity model based on zonal data. Invariably, these methods lead to large uncertainties and unreliable OD inferences.

In this work, we introduce a new OD inference approach using deep neural network (DNN) and a method based on linear regression (LR). We regress for the *probabilities* $\zeta _{ij}$ of an agent going from origin *i* to destination *j*, instead of predicting the *actual number of agents* in the OD matrix. These quantities $\zeta _{ij}$ (also known as “fan-outs”^19^) are assumed to be stationary throughout the period of interest, and are thus always the same. For example, in a bus system, commuters in the morning have some preferred $\zeta _{ij}$, so the fan-outs are constant. But the number of people arriving the bus stops or boarding the buses need not be the same each time. This happens when a next bus arrives relatively quickly after the previous bus has left, with fewer people boarding it. Consequently, simple linear regression can be implemented to obtain $\zeta _{ij}$ from repeated measurements over the period. The actual OD numbers are just the total numbers from the origins (which are easily measurable) multiplied by $\zeta _{ij}$. This approach overcomes the weakness of underdetermined system in previous works, where repeated measurements would correspond to different OD intensities, which cannot be combined. As a result, improvement in prediction accuracy is attained over earlier approaches such as expectation-maximisation and Bayesian inference, which we will demonstrate on a common network in section “Comparison amongst DNNGRU, LR, EM and other traditional methods”.

The assumption of fan-outs being always the same corresponds to real-world conditions. In fact, many papers in the OD literature based their studies on constant fan-outs^1–6^. It is also of practical importance to consider the fan-outs as constant over the period of interest, since fluctuations would mask the overall trend for gleaning meaningful insights. For instance, during a morning commute, we expect commuters travelling from residential areas to commercial areas. This is generally the situation and can be represented by an average fan-out for that period. During off-peak period, this OD distribution tapers off. Hence, various periods would be characterised by its own averaged fan-outs. Additionally, as many existing papers had reasoned^1,4^, non-stationary fan-outs would result in a significantly more complex problem to tackle. Note that this assumption of constant fan-outs have led us to consider general complex networks with separate origin and destination nodes as our models, to signify the idea of morning commute or evening commute.

Next, we go beyond linear regression by training a DNN with supervised learning to predict the OD probabilities $\zeta _{ij}$. In this DNN formulation, we take the total number of agents on each edge at every time step as our input. Our approach is thus applicable to any arbitrary network that connects between a set of origin nodes and a set of destination nodes. In other words, our framework is *network-free*, i.e. directly applicable to any network without requiring explicit modelling and analysis. To illustrate this network-free property, consider the three networks in Fig. 4b–d with different network topologies which all have the same number of nodes and edges. The same deep neural network architecture can be applied, since all it needs are the number of agents on the edges, i.e., the number of input layer nodes of the deep neural network (see Fig. 2) is the same for these three networks. It does not need to know how the edges are connected to which pairs of nodes.

Furthermore, the DNN is composed of gated recurrent units (GRU)^20^ which capture and process the temporal information in our data. The use of GRU is also necessary because our input data is in the form of a time-series, and the same GRU architecture can process time-series input data of arbitrary length. (In contrast, densely connected feedforward DNN would have the number of input nodes dependent on the length of the time-series data.) In comparison, analytical statistical frameworks like the Vardi’s algorithm^7^ and the Bayesian methods^3^ necessitate explicit encoding of the network (referred to as the “routing matrix”, related to the adjacency matrix of a graph) before implementation. Temporal information is not exploited as each datum is treated as being independent from the others.

We harness this DNN framework to study general complex networks with different topologies like lattice, random, and small-world, as well as real-world networks, to glean how network topology affects the accuracy in predicting the OD probabilities. Recently, there is great interest in using deep learning methods to solve problems in applications modelled by complex network, such as predicting the dismantling of complex systems^21^, and on contagion dynamics^22^. While these papers trained deep learning approaches to identify topological patterns on dynamical processes in networks, they have not explored how topology of different complex networks affect the OD prediction accuracies. Incidentally, Ref.^22^ made use of OD information of human mobility to study the spread of COVID-19 in Spain through a complex network model. They compared their DNN approach with a maximum likelihood estimation (MLE) technique and found that the former outperforms the latter. This outcome is analogous to our case because Vardi’s expectation-maximisation algorithm with moments is in fact an MLE method. Moreover, unlike Refs.^21,22^ which implemented graph neural network and graph attention network that made use of convolution of neighbouring nodes to significantly improve performance, we design a GRU architecture (henceforth referred to as DNNGRU) which leverages on temporal information to predict OD probabilities.

Incidentally, whilst indeed there have been research work dealing with linear regression, the main purpose for us to do so here is to provide an analytical analysis with exact analytical results for the expected OD probability distribution so that we can compare with our DNNGRU results. These exact analytical results for the expected values serve as the benchmark result for any machine learning or deep learning models to achieve, hence they are of direct relevance for the purpose of result verification. Our approach here overcomes the underdetermined problem due to the vast training data available for our deep neural network to extrapolate via supervised learning to predict the appropriate OD probabilities. Similar to linear regression, multiple data points by repeated measurements allow for a best fit solution that minimises the mean squared error to be deduced.

DNNGRU is the appropriate neural network architecture for origin-destination inference (as opposed to a non-recurrent neural network (RNN) architecture) because it allows for a time-series of any length to be fed into the same design, since it is an RNN. This allows for transfer learning to be applicable, when building and training DNNGRU models to input time-series of various lengths. Recently, there have been several papers implementing LSTM in the OD prediction problem^23,24^. We have in fact, initially tested it with LSTM but found no significant difference between using GRU versus LSTM. We implemented GRU instead of LSTM, because GRU is less computationally expensive and requires less training time than LSTM. Incidentally, Refs.^23,24^ study the estimation and prediction of OD matrix based on a given city transportation network like for a railway system in Hong Kong and in Poland, respectively. On the other hand, our paper deals with the study on general complex networks to investigate the effects of network topology on the inference of OD probability distribution.

Before concluding our paper, we investigate a simple bus loop system in section “Application: bus loop service”. Here, buses go in a loop to serve commuters in a University campus. We build a simulation programme based on real-world parameters, to generate labelled data where the input are the number of passengers on each bus after the bus leaves a bus stop, with the labels being the OD pairs. We can then generate training data to train our DNNGRU, another dataset for validation during training, as well as a third dataset for testing. In reality, some transportation systems do track the number of passengers onboard the buses or trains at all times, via an automated passenger counter (APC)^25^. This is useful especially for trains to inform new passengers on the load distribution of the train carriages, so that they can move to sections with relatively less people on board. Some transportation operators would even track the OD pairs by the tap in and tap out of each commuter. Each commuter has a unique card with a unique ID, hence these would form the labels for the dataset. However, sometimes such datasets are only released in a partial or coarse-grained manner. For example, Singapore Land Transport Authority releases such data information for hourly aggregated OD pairs which are averaged over the calendar month^26^. Hence, this is a dataset with known labels at a coarse level, but not fully informative towards finer details.

## Results

### Data measurements

Before presenting the linear regression (LR) and deep neural network (DNN) approaches to infer the origin-destination distribution, we elaborate on the data types that are easily measurable within generic real-world complex networks. In our setup, we primarily consider the dataset to be a time-series of *T* time steps where the number of agents are tracked at every single time step. Then, at a single time step, the measurable quantities are: $x_i$ which is the number of agents leaving origin *i*, $y_j$ which is the number of agents arriving at destination *j*, as well as $F_{ab}$ which is the number of agents on the directed edge from node *a* to node *b* of the complex network.

We employ $x_i$ and $y_j$ for LR due to its formulation. For general complex network, LR can only serve as an approximate estimator, unless the network is relatively simple enough to allow for an exact representation. In contrast, conventional algorithms for origin-destination estimation^3,7^ use the number of agents on the edges, $F_{ab}$. Hence, in developing our DNN framework, we will be using only $F_{ab}$ but not including $x_i$ and $y_j$. This mode of data measurement is used in sections “General complex networks” and “Application: bus loop service”.

Nevertheless, in order for us to provide a fair evaluation of our DNN framework as compared to Vardi’s^7^ and Tebaldi-West’s^3^ approaches, we will consider a separate type of measurement in our comparison against these traditional approaches. In this case, we count the number of agents in $x_i$ and $F_{ab}$ over some fixed time interval, i.e. these numbers are aggregated instead of the actual numbers at every time step. Then, *T* such measurements are collected (where this *T* is now the number of independent aggregated measurements, instead of the length of the time series). This means of aggregated measurement is in fact implemented by Vardi and Tebaldi-West, although Vardi’s formulation uses two variables: one counts the number of agents from origin to destination, and the other counts the agents on the edges, which he denotes by $X_i$ and $\vec {Y}$ respectively. To avoid confusion with our notation, we have renamed our $x_i$ as $V_i$ while retaining the use of the Vardi’s vector of aggregated number of agents on all edges $\vec {Y}$ in our formulation. We will use these aggregated variables in our DNNGRU studies as well as that of our LR formulated using these variables described in the Supplemental Material (SM). Note that this mode of data measurement is employed in section “Comparison amongst DNNGRU, LR, EM and other traditional methods”.

### A linear regression approach

**Figure 1:**
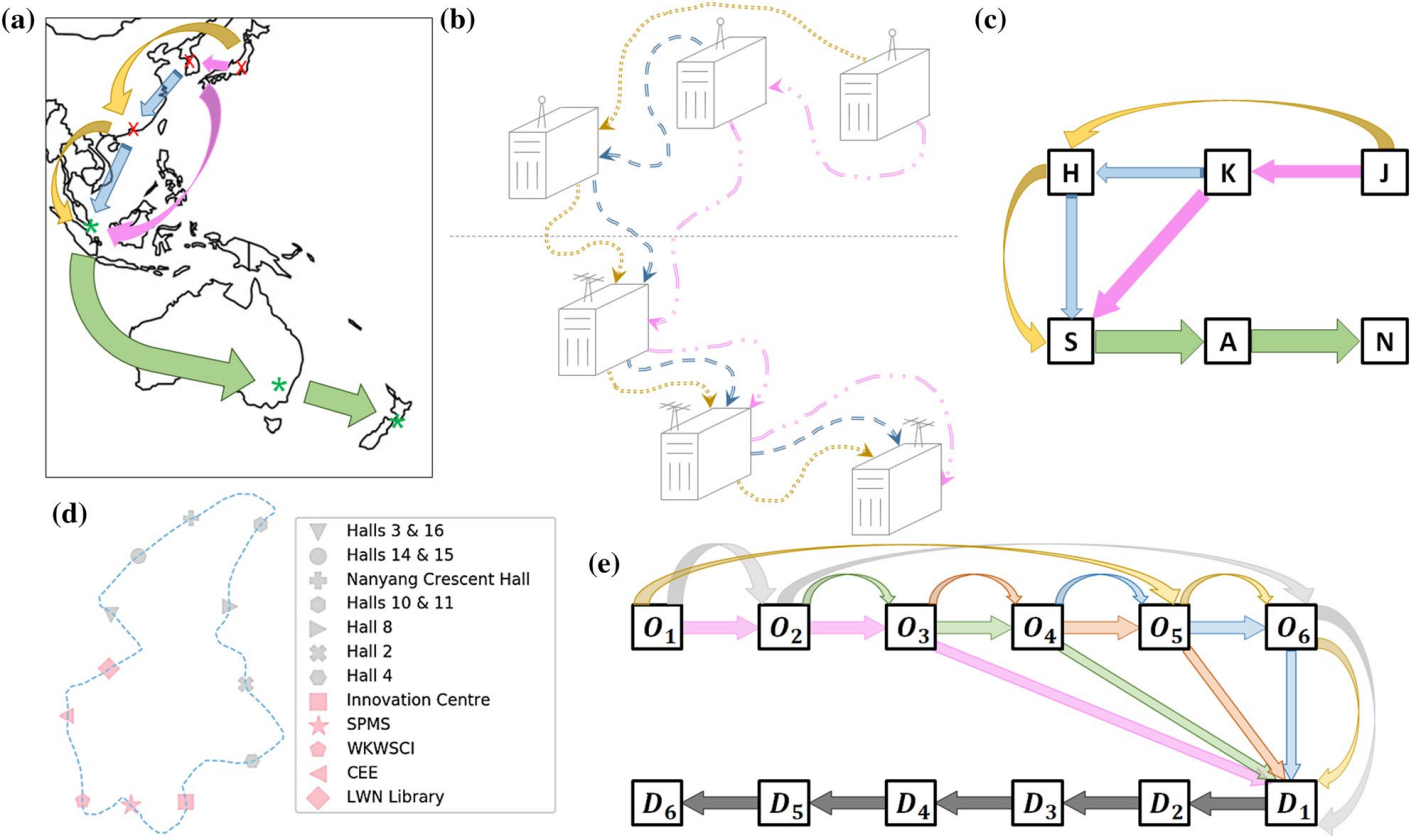
(**a**) Hypothetical example of three origin data servers (red x) connected to three destination data servers (green $*$) in Asia-Pacific. (**b**) Pictorial visualisation of data server connections of (**a**). (**c**) Network representation of (**a**, **b**). (**d**) Blue Route of the NTU campus shuttle bus service^27,28^. (**e**) Network representation of (**d**), where the nodes are linked via a *semi-express* configuration^29,30^. Each of the six coloured arrows represents one semi-express bus picking up commuters from distinct subsets of origins. Subsequently, all buses allow alighting at all destinations $D_1,\ldots , D_6$.

Let us consider a complex network of nodes where agents from an origin node can end up at any destination node in the network. In our context, an edge in the network corresponds to a carrier route that facilitates transfer of agents between the two nodes connected by it. For example, data server hubs are linked through a series of fibre-optic cables (*carriers*). As not all data server hubs are directly connected due to geography, data (*agents*) transfer between a pair of data servers may traverse other data servers along the way, as depicted in Fig. 1a–c. In another example, the bus service in Fig. 1d comprises a loop of 12 bus stops served by buses going around. In other words, buses (*carriers*) must sequentially traverse one bus stop after another to deliver commuters (*agents*) in some fixed order along the prescribed loop.

In a network with $M_O$ origins and $M_D$ destinations, there are $M_O(M_D-1)$ free parameters which quantify the probability distribution of agent-transfer from one node to another. This is because at each of the $M_O$ origins, there are $M_D$ destinations and these probabilities sum to 1. Whilst we do not know where each agent goes, we can write down this general multivariate linear system:

1 $$\begin{aligned} y_j=\sum _{i=1}^{M_O}{\zeta _{ij}x_i},\text { for }j=1,\ldots ,M_D, \end{aligned}$$

subject to the constraints $\displaystyle \sum \nolimits_{j=1}^{M_D}{\zeta _{ij}}=1$, for $i=1,\ldots ,M_O$. Here, $x_i$ are the number of agents the carrier picks up from origins $i=1,\ldots ,M_O$ whilst $y_j$ are the number of agents that carrier delivers at destinations $j=1,\ldots ,M_D$. The sought after quantities are $\zeta _{ij}$, denoting the OD probabilities of agents from *i* to *j*. As Eq. (1) does not account for agent-transfer through the edges with different travelling paths between the same origin and destination, it serves basically as an approximation model.

Assuming that $\zeta _{ij}$ are stationary and hence independent of time, repeated measurements will yield different $x_i$ and $y_j$ leading to an overdetermined set of equations given through Eq. (1). The OD coefficients $\zeta _{ij}$ can then be deduced by linear regression (LR), given dataset $(x_i,y_j)$ to be fitted^31^. In other words, we analytically solve for $\zeta _{ij}$ through the minimisation of the mean squared error. As LR gives coefficients $\zeta _{ij}\in {\mathbb {R}}$, there are occasions when $\zeta _{ij}<0$. We handle this by minimally shifting the entire $\zeta _{ij}$ to make all $\zeta _{ij}\ge 0$, and then normalise them so that the $M_O$ constraints below Eq. (1) are satisfied. This formulation constitutes a linear framework of OD estimation modelled by a complex network that relates to a particular system of interest. Depending on the system under examination, the variables $x_i$ and $y_j$ are determined from the relevant measured empirical data. We have compared our LR approach with quadratic programming where the optimisation is performed by imposing the additional constraint that $\zeta _{ij}$ is non-negative. While quadratic programming is observed to perform consistently better than LR at small data size, LR takes a significantly shorter time to reach the solution relative to quadratic programming. Both approaches nonetheless converge to the same solution when sample size increases.

In the case of the bus system, publicly accessible information such as the number of people on buses and the duration buses spend at bus stops^26^ can be used to provide $x_i =$ number of people boarding and $y_j =$ number of people alighting the associated bus. In the context of data servers, $x_i$ and $y_j$ are deducible from the traffic load passing through the fibre-optic cables carrying packet bits since increased/decreased load is due to $x_i$/$y_j$ from/to a data server.

### DNN with supervised learning

**Figure 2:**
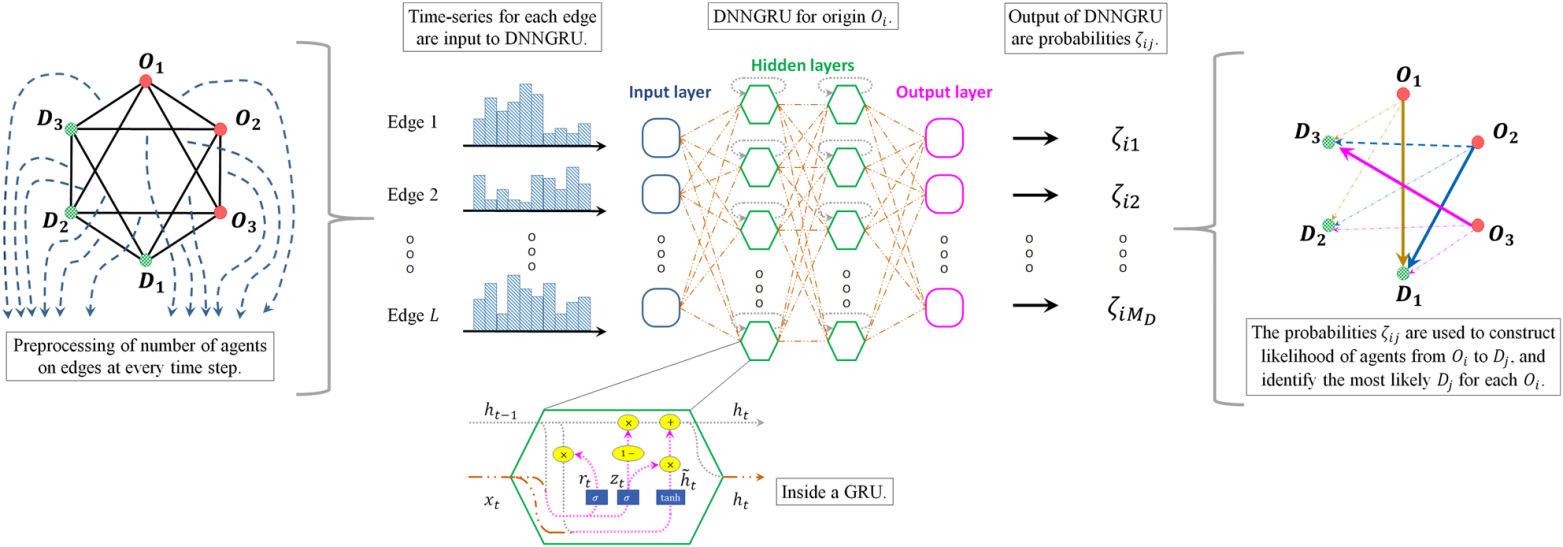
Number of agents $\mathscr {N}_{st}$ from each edge *s* of a complex network are measured at a time instant *t*. After preprocessing them into time-series of length *T* for every edge, the inputs $\mathscr {N}_{st}$ (where $s=1,2, \ldots , L$, and $t = 1,2,\ldots , T$) are fed into a DNNGRU and trained by supervised learning to predict $\zeta _{ij}$. Separate DNNGRUs are trained for each origin $O_i$ and each time-series length *T*. From the outputs $\zeta _{ij}$, we construct the likelihood of agents going from $O_i$ to $D_j$, as well as identify the most likely destination from each origin node.

The main idea of our approach is to determine the OD probabilities $\zeta _{ij}$ from easily accessible or directly measurable information, specifically the number of agents on the edges of the complex network. This ability to infer $\zeta _{ij}$ is particularly important when it is impossible to measure $\zeta _{ij}$. For example, it is extremely challenging for investigators to figure out the tracks of nefarious hackers/fugitives who would doubtlessly obfuscate their direct transfer of data packets across various data servers. Such problems like commuters in transportation systems as well as fugitive hunting motivate the inference of the most likely destination of agent transfer, from an origin, so that we can better improve service and connectivity or to better locate a target’s whereabouts. We will thus measure the prediction accuracy of our algorithms developed in this paper in predicting the most popular destination with respect to an origin.

In order to deal with these more general situations, we develop a DNN architecture composed of GRU (i.e. DNNGRU) to output $\zeta _{ij}$ by supervised learning, where training may be performed on real-world datasets which are amply available. In addition, labelled data can also be generated through simulation from systems with known characteristics for DNN to generalise from them to new unseen inputs. Figure 2 gives an overview on the approach of this work, where information of the number of agents on the edges (e.g. number of people on buses after leaving bus stops) are passed into DNNGRU to infer OD probabilities of the network.

Our DNN can take as input a more generic form of the dataset with $(x_i,y_j)$ implicitly contained, as compared to LR described in the previous subsection. It thus encompasses a more general data and model structure of the system-under-study such that the actual mechanism of the carriers may be complex and nonlinear. Supervised learning then allows our DNN to learn directly and more generally from labelled $\zeta _{ij}$. Furthermore, we adopt a DNN with sigmoid activation to naturally output the correct range of $\zeta _{ij}\in [0,1]$. An important characteristic of our designed DNN architecture is the incorporation of GRU to enable inference from time series. The GRU is a recurrent unit with hidden memory cell that allows for information from earlier data to be combined with subsequent data in the time-series. This is crucial as the dataset $(x_i,y_j)$ may carry temporal information. For instance, buses recently picking up commuters would leave less people for subsequent buses, hence cascading effects (like bus bunching, overtaking, load-sharing^27,32^) induce deviations from Eq. (1) which ignores temporal correlation as it treats each datum independently.

### Comparison amongst DNNGRU, LR, EM and other traditional methods

**Figure 3:**
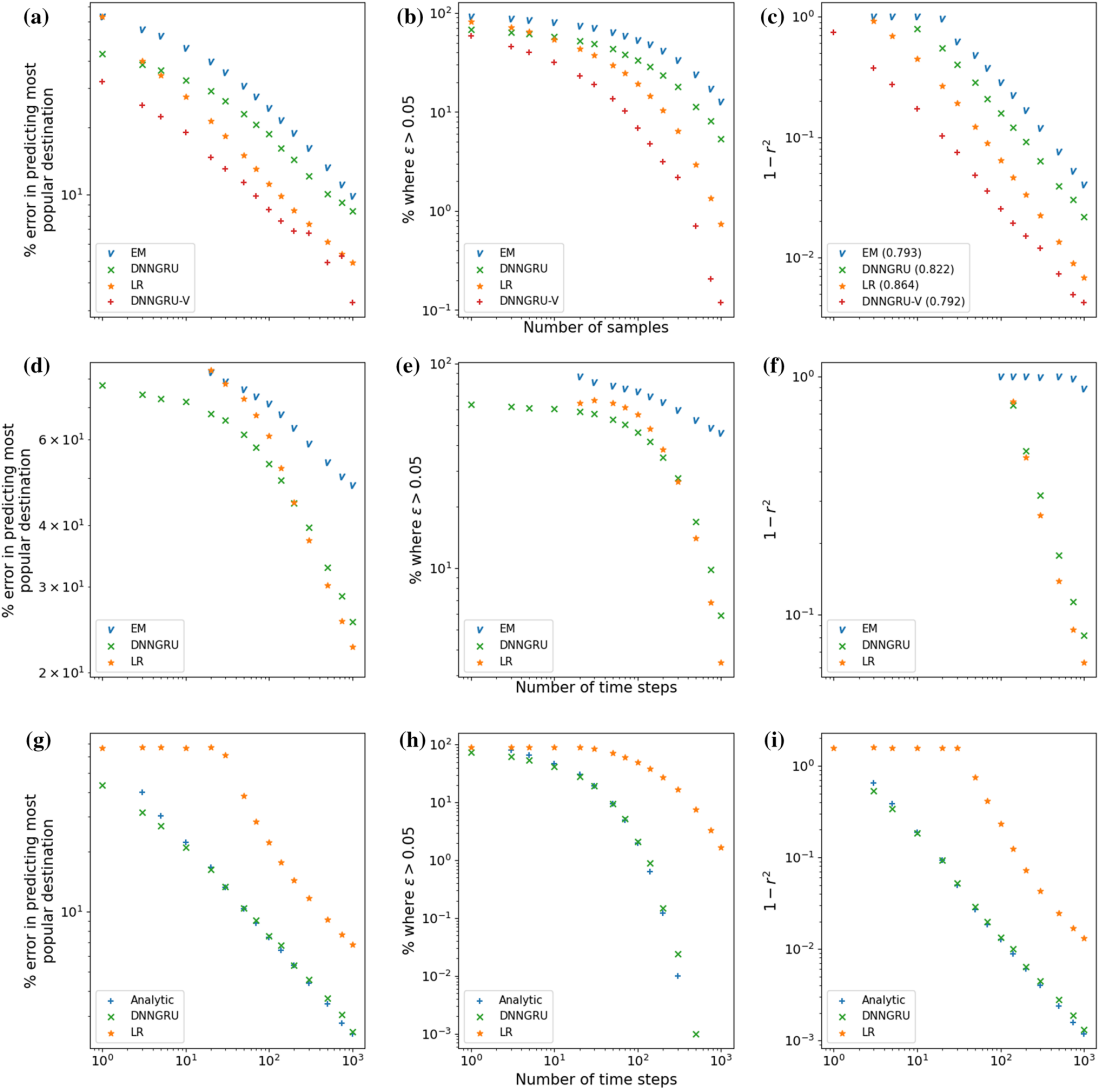
(**a**–**c**) Comparison amongst DNNGRU and Vardi’s EM algorithm for Vardi’s network^7^ as well as LR and DNNGRU-V with additional data on the total number of agents from the origin nodes. Shown in parentheses of the legend in (**c**) are the exponents of a power law fit. (**d**–**f**) Corresponding comparison amongst DNNGRU, LR, and Vardi’s EM algorithm^7^ for the loop in Fig. 4a. (**g**–**i**) Corresponding comparison amongst DNNGRU, LR and an analytical averaging for a lattice with $M_O=M_D=3$ in Fig. 4g. Here, LR in Eq. (1) is only an approximation as it does not track the exact number of $y_j$. In (**h**), note that for $T>500$, DNNGRU and the analytic treatment have $0\%$ error in predicting $\zeta _{ij}$ to be within 0.05 from the true value. Therefore, this would be $-\infty$ on the log-scale, which is not shown.

We directly compare our DNNGRU and LR approaches with the traditional Vardi’s expectation-maximisation (EM) algorithm, Willumsen’s entropy maximisation, as well as Bayesian method on Vardi’s network as a testbed^7^. For this purpose, we adapt the generation of data according to the approach of Vardi: The number of agents on the edges are measured over an extended time period^3,7^. This would be a setup where a counter tracks the overall number of agents that passes through an edge throughout the entire day, for instance. In this case, samples from various days are independent, with the number of people $X_k$ generated for each origin-destination pair being a Poisson random variable with a fixed mean $\lambda _k$. Vardi’s network has four nodes, and consequently 12 OD components in $\vec {X}$ (see Methods for a review of Vardi’s EM algorithm and notations).

We generate datasets in the following manner: For each dataset, each $\lambda _k$ is an integer chosen from [1, 20]. Then, an $\vec {X}$ is drawn, with $\vec {Y}$ computed via Eq. (4). Here, $\vec {Y}$ is the vector of number of agents on the edges of the network. The $\zeta _{ij}$ are computed from appropriately normalising $\vec {\lambda }$ with respect to each origin node. In other words, the ground truth of $\zeta _{ij}$ are: $\zeta _{11}=\lambda _1/(\lambda _1+\lambda _2+\lambda _3)$, etc. Therefore, for each dataset, we have $\zeta _{ij}$ and $\vec {Y}$. The goal is to infer $\zeta _{ij}$ from $\vec {Y}$. We collect *T* samples of $\vec {Y}$ in each dataset for that specific $\zeta _{ij}$, and this provides the input for DNNGRU, as well as Vardi’s algorithm. More datasets can be generated by resetting $\vec {\lambda }$. For LR to be applicable as an exact model, we need to input an additional piece of information, viz. the total number of people originating from each origin node, $\vec {V}$. The exact solution of $\zeta _{ij}$ by LR is given in SM. This additional information is also provided to train an alternative DNNGRU, which we call “DNNGRU-V” to distinguish it from the version that does not include $\vec {V}$ as its input.

Separate DNNGRUs (as well as DNNGRU-Vs) are trained to output $\zeta _{ij}$ for each *i*, so a network with $M_O$ origin nodes has $M_O$ DNNGRUs. Furthermore for each *i*, we let $T=1, 3, 5, 10, 20, 30, 50, 70, 100, 140, 200, 300, 500, 750, 1000$, and apply transfer learning (see Methods) to separately train different DNNGRUs for different *T*. Training comprises 250 k datasets (where $\zeta _{ij}$ has been randomised), with another 10 k as validation during training. Additionally, 50 k datasets (with randomised $\zeta _{ij}$) are for testing. Note that we can train a single DNNGRU for all origin nodes, and we have done that. It turns out that having separate DNNGRUs exclusively for each origin node would improve the accuracy as compared to a single DNNGRU for all origins. Hence we only consider separate DNNGRUs. Later on when dealing with general complex networks in section “General complex networks”, such separate DNNGRUs allow us to study the effect of topology on accuracy with respect to each origin.

After obtaining these probabilities $\zeta _{ij}$, we determine the most popular destination *j* with respect to origin *i* by selecting the corresponding *j* with the highest probability $\zeta _{ij}$. We also determine how accurate is the prediction of $\zeta _{ij}$ with respect to the true value, by two different measures, viz. the difference $\varepsilon =|\zeta _{ij, predicted} - \zeta _{ij, true}|$, as well as the coefficient of determination $r^2$ (see Method) for the straight line fit of $\zeta _{ij,predicted}=\zeta _{ij,true}$. Figure 3a shows the percentage error *E* in predicting the most popular destination, versus the number of samples *T*. Figure 3b shows the percentage of the predictions where $\varepsilon >0.05$, versus *T*. Figure 3c shows $1-r^2$ for the straight line fit of $\zeta _{ij, predicted}=\zeta _{ij, true}$, versus *T*.

Evidently, DNNGRU outperforms EM with the usual data of number of agents on all the edges. We also observe LR to perform better than DNNGRU and EM, which results from the exploitation of the additional data of $\vec {V}$. Note that $\vec {V}$ is easily obtainable for example by placing a counter that tracks every agent that leaves that node, without actually knowing where the agents are going. The use of $\vec {V}$ has also resulted in improvement in the outcome of DNNGRU-V, where it is observed to have superior performance relative to LR. DNNGRU (and DNNGRU-V) is advantageous here by benefiting from the many weights and biases to be tuned from supervised learning with a deep neural network. Basically, the enhanced performances of DNNGRU and DNNGRU-V over Vardi’s algorithm and LR, respectively, arise from the gains accrued by learning from data. Nonetheless, the performance of DNNGRU-V would converge to that of LR in the asymptotic limit of large *T* since LR gives exact solution (see Fig. 1a SM). On the other hand, LR shows better performance compared to EM due to its lower modelling uncertainties from fewer underlying assumptions. As for entropy maximisation and Bayesian inference, the results are below par and hence not displayed. The latter two methods have not addressed the underdetermination problem and consequently are still burdened by the high uncertainty of their results. See SM for more details.

Incidentally, results corresponding to Fig. 3c where the predictions are the actual OD intensities instead of $\zeta _{ij}$ are shown in Fig. 1b of SM. To predict the actual OD intensities, we just multiply the predicted probabilities $\zeta _{ij}$ with the corresponding average total number of people at the origins. This is fully in accordance to how Vardi intended to apply his expectation-maximisation algorithm. In terms of the actual OD intensities, again LR, DNNGRU, DNNGRU-V all outperform EM.

**Figure 4:**
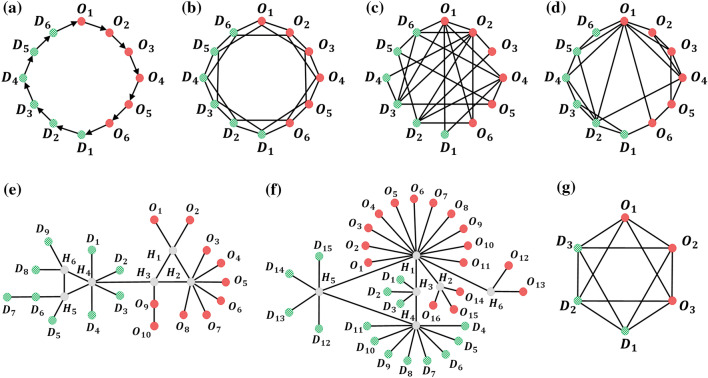
(**a**) Directed loop, with 11 ADE. (**b**) Lattice, with 38 ADE. (**c**) Random, with 32 ADE, (**d**) Small-world, with 24 ADE, (**e**) VinaREN, with 24 ADE, (**f**) MYREN, with 40 ADE, (**g**) smaller lattice, with 18 ADE. In each network, $O_i$ (red) are origins, $D_j$ (green) are destinations. (**a**–**d**) have 6 origins and 6 destinations, whilst (**e**) has 10 origins and 9 destinations with 6 intermediate nodes (grey), (**f**) has 16 origins and 15 destinations with 6 intermediate nodes (grey). The smaller lattice in (**g**) has 3 origins and 3 destinations. (**b**–**d**) all have 24 edges (48 directed edges, since each edge represents both directions), so they differ on their topologies being a lattice, random, or small-world network.

A performance analysis and comparison between DNNGRU (without $\vec {V}$), LR, and EM have also been performed for a loop network (Fig. 4a) with the same outcome observed. Details are given in Methods, with corresponding results displayed in Fig. 3d–f. The data generation here is not based on measuring the agents over a specified time interval, as prescribed by Vardi’s network^7^. Instead, it is based on the realistic flow of agents from one edge to the next edge, which we elaborate further in the next subsection on general complex networks. The point here is, DNNGRU and LR are superior to Vardi’s EM algorithm. All three methods receive the same information of the number of agents on the edges at every time step, over *T* time steps.

### General complex networks

**Figure 5:**
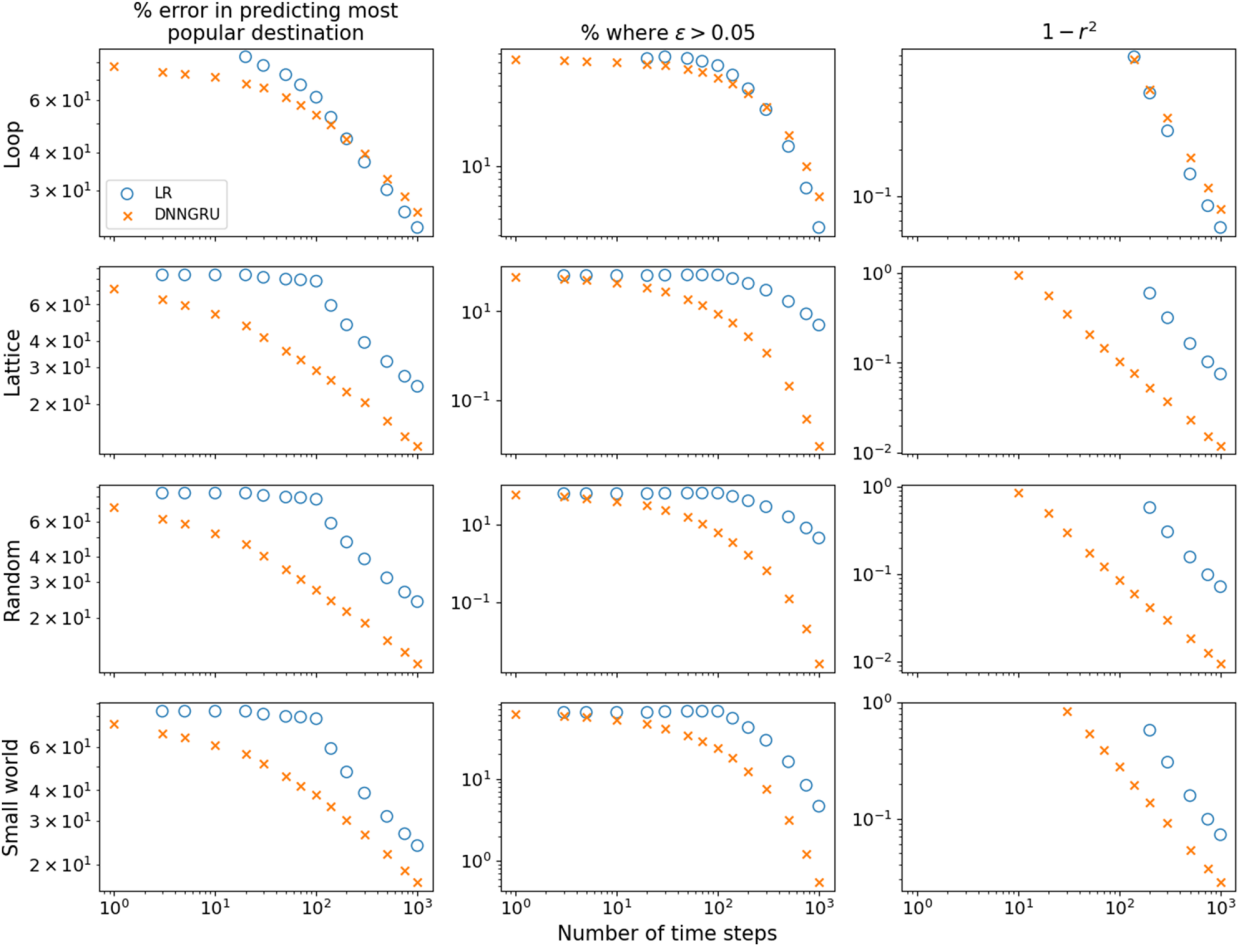
DNNGRU outperforms LR in the lattice, small-world and random networks with the same number of nodes and edges, as LR serves as an approximation. For the loop, LR is an exact model and asymptotically matches the performance of DNNGRU.

**Figure 6:**
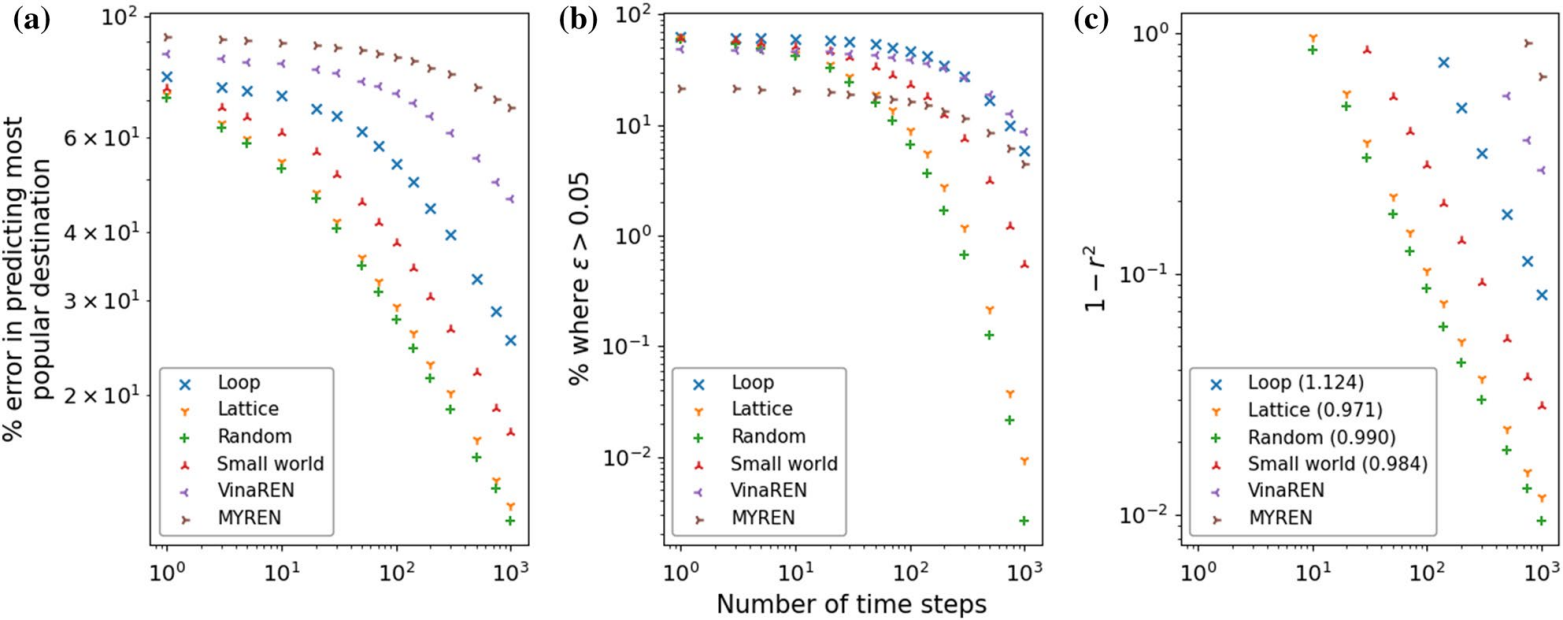
(**a**) The percentage error in predicting the most popular destination with respect to an origin, for various networks. Each plot point is the result of supplying as input data the last *T* time steps of the number of agents on every ADE of the network. (**b**) The corresponding plot showing the percentage of predictions where $\varepsilon >0.05$. (**c**) The corresponding plot showing $1-r^2$, with $r^2$ being the coefficient of determination of fitting $\zeta _{ij, predicted}=\zeta _{ij, true}$. Shown in brackets in the legends of (**c**) are the exponents for the respective best fitted power laws. See Fig. 2 in SM for corresponding plots with lag of $l=10$ to traverse an edge.

**Figure 7:**
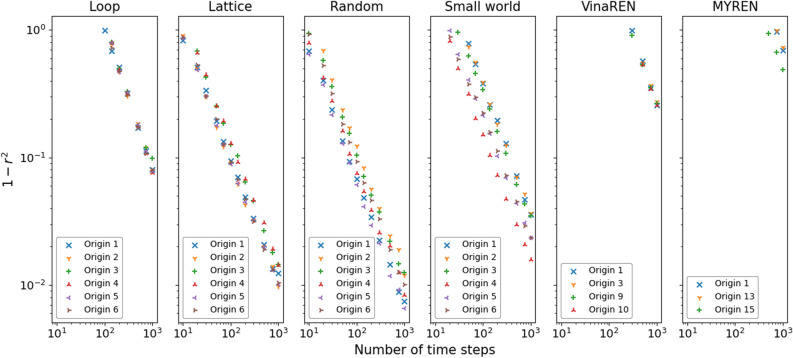
In contrast to Fig. 6 where each plot point is the average result over all origins, here are the corresponding results with respect to each origin, for $1-r^2$. For VinaREN and MYREN, many origin nodes are equivalent. Hence, there are only 4 distinct types of origins for VinaREN and 3 distinct types of origins for MYREN. See Figs. 3–5 in SM for the plots of *E* and $\varepsilon$, as well as with lag $l=10$.

**Figure 8:**
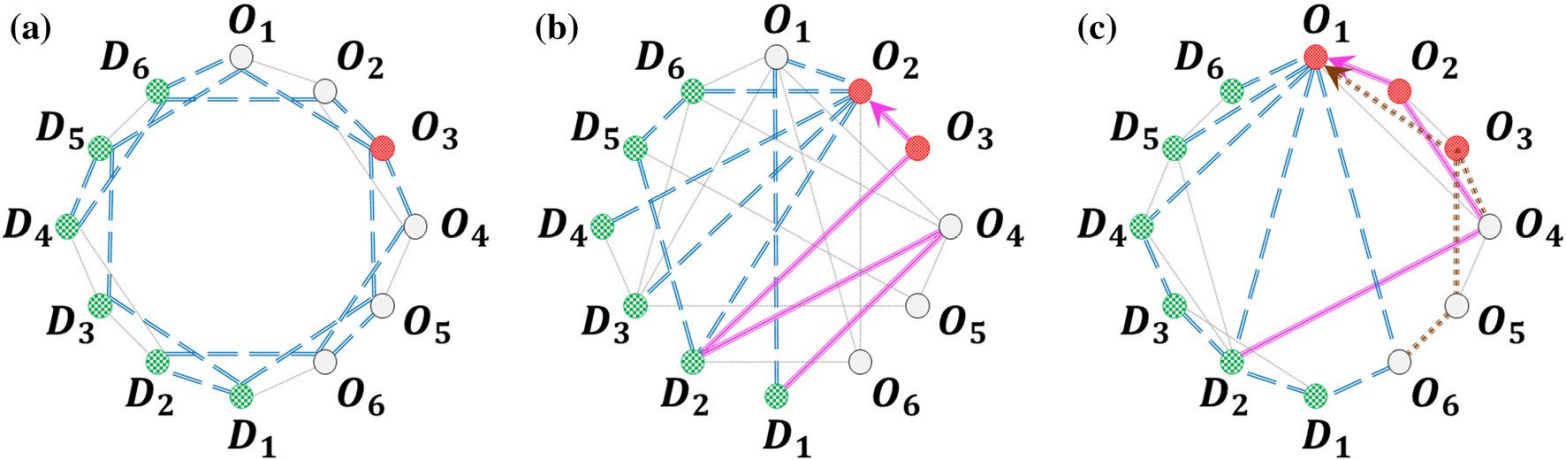
The lattice, random and small-world networks from Fig. 4b–d. Here, we highlight the origin nodes that are relatively worst in predicting the most popular destination, compared to other origin nodes. (**a**) (lattice) $O_3$ and $O_4$ are the least accurate. Shown here is with respect to $O_3$, which is the furthest from all destination nodes. So, $O_3$ has many edges which overlap with the edges used by others. (**b**) (random) Shown here are the edges used by $O_2$ to get to all destination nodes, with $O_3$ being a *parasitic* origin node. This is because whilst $O_3$ uses $O_3\rightarrow D_2$ and $O_3\rightarrow D_2\rightarrow O_4\rightarrow D_1$, it otherwise overwhelmingly goes to $O_2$ and traverses all the edges used by $O_2$ to get to the destination nodes. (**c**) (small-world) Shown here are the edges used by $O_1$ to get to all destination nodes, with $O_2$ and $O_3$ both being parasitic nodes as they overwhelmingly go to $O_1$ and traverse all the edges used by $O_1$ to get to the destination nodes.

Consider a network with $M_O$ origin nodes and $M_D$ destination nodes. At each time step, origin *i* can generate any number from 1 to *P* agents each assigned to go to destination *j* with probability $\zeta _{ij}$. Then, the agents propagate to subsequent edges in their paths as the time step progresses. Agents take the shortest path via edges of the network. If there are many shortest paths, one is randomly chosen (out of a maximum of *n* pre-defined shortest paths). We consider two versions, viz. when an agent traverses an edge, it spends one time step before leaving that edge to another edge or arriving at its destination (without lag); or it spends *l* time steps on the edge before proceeding (with lag). The number of agents on each edge is recorded every time step. The system is allowed to evolve, and the last *T* time steps are taken to train a DNNGRU.

We consider various such complex networks in our study (Fig. 4). As presented in the previous subsection where we compared with Vardi’s EM algorithm, Fig. 4a is a directed loop with $M_O=M_D=6$. Then, we study three networks: Fig. 4b lattice, Fig. 4c random, Fig. 4d small-world, with the same number of origins and destinations ($M_O=M_D=6$) as well as edges (24 edges or 48 directed edges). These three networks provide a basis for comparison across different network topologies. Subsequently, we adopt two real-world internet networks^33^: Fig. 4e VinaREN, and Fig. 4f MYREN, to investigate the effects of real network topologies. The internet VinaREN and MYREN in Fig. 4e, f have $M_O=10,M_D=9$ and $M_O=16,M_D=15$, respectively. Note that the directed loop in Fig. 4a is also a real-world network of interest, and is studied in greater detail in section “Application: bus loop service”. In our study, we have set $P=10$, $n=4$, and $l\in \{1,10\}$. Also, the number of agents originating from each origin is changed every 20 time steps to introduce stochasticity and variation, though $\zeta _{ij}$ is kept constant throughout each dataset. The adjacency matrices for the networks in Fig. 4b–d and their set of shortest paths used from $O_i$ to $D_j$ are summarised in SM. Those for the other networks are deducible in a straightforward manner from Fig. 4. On top of these complex networks, we find it instructive to study a small lattice with $M_O=M_D=3$ (Fig. 4g), where we carry out an analytic treatment and compare against DNNGRU and LR.

We refer to the number of *active directed edges* (ADE) as those links that are actually being used, i.e. forming part of the shortest paths from origins to destinations. The number of ADE for the networks in Fig. 4a–g are, respectively: 11, 38, 32, 24, 24, 40, 18. More ADE implies more information being tracked. Let the number of agents on every ADE at every time step form the feature dataset to be fed into the input layer of a DNNGRU, lasting *T* time steps (c.f. Fig. 2). Unlike the previous subsection where we tested on Vardi’s network, information of $\vec {V}$ is not supplied to DNNGRU, i.e. we no longer consider DNNGRU-V in the rest of this paper.

#### A small lattice

In order to gain a deeper understanding on the performance of LR and DNNGRU, we employ a small lattice (Fig. 4g) that is amenable to analytical treatment. The analytical treatment essentially tracks the paths of agents through the various edges, to arrive at a set of equations which exactly solves for all the unknown variables (elaborated in SM). Specifically, as the agents propagate from an origin node of the small lattice, those who are destined for the same end node may traverse different paths, and the travelling pattern of the agents can be modelled analytically. Nonetheless, there is still stochasticity in the analytical treatment due to the stochastic nature of agents spawning at the origins. And with the evaluation of $\zeta _{ij}$ based on the ratio of two integer number (of agents), there can still be uncertainties in the prediction of $\zeta _{ij}$. Note that the analytical treatment is achievable here due to the small lattice having few nodes with a relatively high number of edges. On the other hand, LR as given by Eq. (1) is only an approximation as it does not track the correct number of $y_j$. Thus, we observe the better performance of the analytical treatment over LR in Fig. 3g–i. DNNGRU matches with the analytical results, but appears to be slightly inferior in the asymptotic limit with large *T*, perhaps due to the optimisation of DNNGRU not finding the absolute best minimum of the loss.

#### Topological effects of the complex networks

Let us concern ourselves with the three networks: lattice; random; and small-world (Fig. 4b–d), which have the same number of origin and destination nodes, and also the same number of edges. Due to the different topologies of these networks, they have distinct ADE and thus diverse shortest paths that the agents could travel from the origin node to destination node. As DNNGRU learns a model that takes these different shortest paths of the agents into account while LR does not, DNNGRU outperforms LR in prediction accuracy as shown in Fig. 5. (Note that LR is an exact model for the loop in Fig. 4a, and we see convergence between LR and DNNGRU for large *T* in the top row of Fig. 5).

Figure 6 shows results of DNNGRU applied to the networks in Fig. 4a–f, so we can compare the performances across various networks on the same plots. We examine how topology influences the performance of the three networks with equal number of nodes and edges using DNNGRU, i.e. those in Fig. 4b–d. It turns out that the small-world topology performs the worst, given the same number of nodes and edges, because many shortest paths prefer taking the common “highway edges”, the very property that makes it small-world. The ramification of this is its lesser ADE (at 24) which carries less information than the lattice or random networks with greater ADE. This observation is, however, inconsistent with the fact that the random network which has a lesser ADE of 32 outperforms the lattice network with a higher ADE of 38. The deeper reason is revealed by looking into the performance variation across different origin nodes within the network.

Recall that each origin node *i* is trained with its own DNNGRU to exclusively predict $\zeta _{ij}$ for that *i*. Figure 7 reports the results for $1-r^2$ versus *T* with respect to each particular origin node. Corresponding results for the percentage errors of predicting the most popular destination as well as those for $\varepsilon >0.05$ are given in SM.

Since the lattice, random and small-world networks have origin nodes with different topological properties, the agents could have alternative pre-defined shortest paths to take. Consequently, different network topologies do indeed lead to different origin nodes performing better than others. We can determine the relatively worst performing origin nodes in these networks, by examining the graphs in Fig. 7. For the lattice, origin nodes $O_3$ and $O_4$ (which are equivalent, due to the symmetry of the lattice) have a relatively higher error compared to the other origin nodes. For the random network, the worst performing origin nodes are $O_2$ and $O_3$ with $O_6$ also relatively poor, whilst those for the small-world network are $O_1$, $O_2$ and $O_3$.

Accuracy in inferring $\zeta _{ij}$ relies on the ability to disentangle the individual *i*, *j* components from the combined information when they add up on the shared edges. The lattice in Fig. 8a shows how $O_3$ (and $O_4$, by symmetry) tends to be inferior compared to the rest, because it is the furthest away from all destination nodes. This means that it has to traverse relatively more edges, and consequently overlap with more edges used by other origin nodes.

Another way an origin node can become inferior is due to a *parasitic origin node* that taps on it to get to the destinations. This is obvious in the random and small-world networks, as depicted in Fig. 8b, c. For the random network, $O_3$ has essentially all its paths relying on all those used by $O_2$. This makes both of them suffer slightly worse performance, since it is more difficult to ascertain the $\zeta _{ij}$ to be attributed to which of them. Other origin nodes have more diverse paths, allowing for more information available to infer their own $\zeta _{ij}$. Similarly for the small-world network, $O_2$ and $O_3$ are parasitic origin nodes with majority of their paths going via those of $O_1$, resulting in all three of them being slightly inferior than other origin nodes.

Let $E_{ij}$ be the total number of edges that origin nodes $O_i$ and $O_j$ would overlap, when getting to all destinations. We summarise the values of $E_{ij}$ for these three networks in Table 1. Generally, origin nodes that have greater overlap, i.e. larger $E_{ij}$, would tend to be less accurate. These can be made more precise by encapsulating them into an index $\chi _i$ for each origin node $O_i$ which quantifies the relative degree of overlap amongst them:

2 $$\begin{aligned} \chi _i&=\frac{1}{E_i(M_O-1)}\sum _{i\ne j}{E_{ij}^2}. \end{aligned}$$

In Eq. (2), $E_i$ is the number of edges that are involved in getting from $O_i$ to all destinations. The division by $M_O-1$ serves to make $\chi _i$ as an averaged quantity over all the overlapping origin nodes (minus one to exclude itself). The division by $E_i$ is to normalise as nodes with more edges would tend to proportionally overlap more with other origins’ edges. The rationale for taking the sum of squares of $E_{ij}$ (as opposed to just the sum of $E_{ij}$) is because an edge overlap involves two such origin nodes $O_i$ and $O_j$, hence $E_{ij}$ should arguably appear as two factors. The squaring would also more greatly penalise higher overlapping numbers as compared to fewer overlaps. This is especially critical for an origin node with its parasitic companion which together share a larger amount of edges $E_{ij}$, and which would raise both their indices values $\chi _i$ and $\chi _j$ by $\sim E_{ij}^2$.

**Table 1:** Crosstables for the lattice, random and small-world networks from Fig. 4b–d.

Node	$O_1$	$O_2$	$O_3$	$O_4$	$O_5$	$O_6$	$\chi _i$
Lattice							
$O_1 (11)$	–	6	7	3	3	0	1.87
$O_2 (16)$	6	–	8	10	4	3	2.81
$\mathbf{O_3 (16)}$	**7**	**8**	**–**	**8**	**10**	**3**	**3.58**
$\mathbf{O_4 (16)}$	**3**	**10**	**8**	**–**	**8**	**7**	**3.58**
$O_5 (16)$	3	4	10	8	–	6	2.81
$O_6 (11)$	0	3	3	7	6	–	1.87
Random							
$O_1 (12)$	–	4	2	5	2	5	1.23
$\mathbf{O_2 (8) }$	**4**	**–**	**6**	**3**	**0**	**5**	**2.15**
$\mathbf{O_3 (10) }$	**2**	**6**	**–**	**3**	**1**	**5**	**1.50**
$O_4 (14)$	5	3	3	–	6	3	1.26
$O_5 (9)$	2	0	1	6	–	0	0.91
$\mathbf{O_6 (10) }$	**5**	**5**	**5**	**3**	**0**	**–**	**1.68**
Small-world							
$\mathbf{O_1 (9)}$	**–**	**9**	**9**	**5**	**5**	**5**	**5.27**
$\mathbf{O_2 (12)}$	**9**	**–**	**10**	**6**	**6**	**5**	**4.63**
$\mathbf{O_3 (14)}$	**9**	**10**	**–**	**6**	**8**	**5**	**4.37**
$O_4 (9)$	5	6	6	−	8	3	3.78
$O_5 (15)$	5	6	8	8	–	5	2.85
$O_6 (8)$	5	5	5	3	5	–	2.73

The numbers in parentheses in the left most column of each crosstable indicate the total number of edges $E_i$ involved for that node to get to all destinations. These crosstables show the number of edges $E_{ij}$ that origin node $O_i$ overlaps with those of origin node $O_j$, when getting to any destination. Rows in bold show the poorer or poorest performing nodes for that network, as deduced from Fig. 7. As revealed by the crosstables, these correspond to large values of $\chi _i$, signifying greater overlaps.

As revealed in Table 1, larger values of $\chi _i$ would correspond to that origin node $O_i$ being less accurate (or larger error) relative to other origin nodes within that network, as deduced from Fig. 7. Incidentally, this index $\chi _i$ also appears to correspond to the random network performing better than the lattice, with the small-world network as the worst. The average values $\bar{\chi }$ of $\chi _i$ for these networks (random, lattice, small-world) are 1.46, 2.75, 3.94, respectively. This was, in fact, already deduced from Fig. 6 earlier. Intriguingly, the average index $\bar{\chi }$ explains why the random network with 32 ADE manages to outperform the lattice with 38 ADE: The former’s average $\bar{\chi }$ is smaller which indicates less overlapping of edges used between the origin nodes. Thus, by accounting for the overlapping of edges as a measure of how hard it is to disentangle which agents are from which origins to which destinations, we obtain an indicator of which origin nodes are easier to predict the OD probabilities. On the other hand, we are able to verify this empirically using the model-free DNNGRU on each origin node (as displayed in Fig. 7).

#### No overlap, partial overlap, superhighway networks

**Figure 9:**
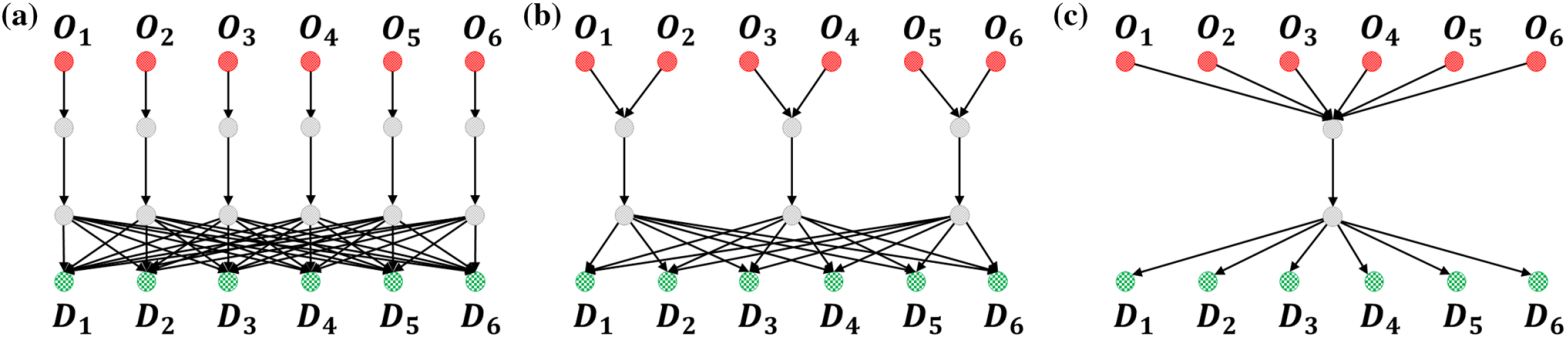
Three networks with 6 origins and 6 destinations, with differing levels of overlap. (**a**) No overlap. (**b**) Partial overlap. (**c**) Superhighway.

In this subsection, we provide a direct illustration of the effects of topology and sharing of edges on the accuracy of predicting OD information through a study of idealised networks as shown in Fig. 9. Here, three network with six origins and six destinations are considered. The first has no overlap, so inferring $\zeta _{ij}$ from the edge counts does not contain the uncertainties of the origin or destination on which the agent traverses, though there is still stochasticity involved since agents choose their destinations probabilistically. The second network has partial overlap, since two origin nodes share a common edge. The inference of $\zeta _{ij}$ can be achieved by simple linear regression involving the pair of nodes, based on Eq. (1). Finally, the third network has all six origin nodes sharing a common superhighway edge. Similar to the second network, here linear regression is directly applicable with all six origin nodes.

**Figure 10:**
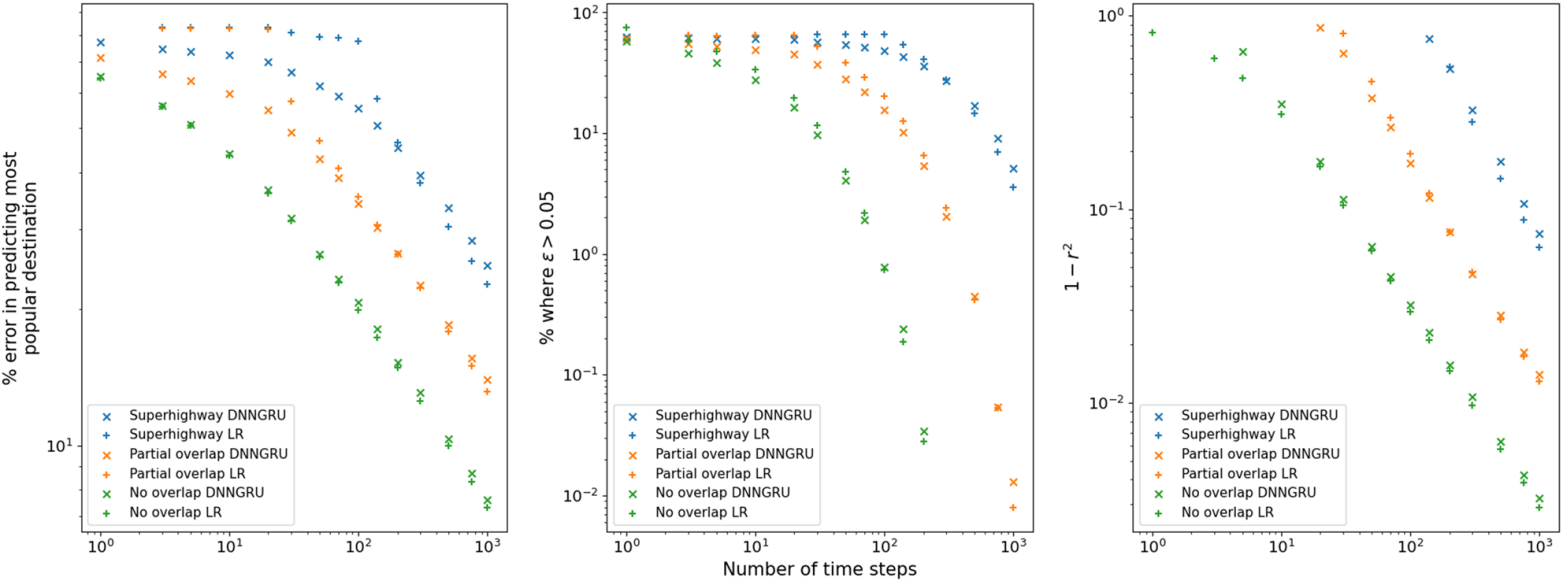
Performance comparison between DNNGRU and LR for the three networks in Fig. 9.

The results are shown in Fig. 10 . It is evident that no overlap is the easiest to infer $\zeta _{ij}$, with the superhighway network being the hardest. Because LR provides an exact formulation of the three networks, it consistently performs the best given sufficient data. In all the three networks, we observe that accuracy improves in a power law manner with more data, with DNNGRU matching the results of LR. We can calculate $\bar{\chi }$ for each of these networks. The index value for the no overlap network is zero, since $E_{ij}=0$ for all pairs of origins. For the partial overlap network, the sum of $E_{ij}^2$ in Eq. (2) is only $7^2$ as each origin only overlaps with one other origin. Finally, the sum of $E_{ij}^2$ for the superhighway network is $5\times 7^2$ since each origin overlaps with five other origins. Consequently, $\bar{\chi }=0,1.225,6.125$, respectively for the no overlap, partial overlap and superhighway networks. This again shows $\bar{\chi }$ as a measure of the degree of shared edges, with higher values signifying lower accuracy. Nevertheless, note from Fig. 10 that increasing the data size would improve the prediction accuracy in a power law manner in all cases, even for a superhighway network where all information pass through a common edge.

#### Real-world complex networks

Let us now consider the topology of three real-world complex networks: the directed loop, VinaREN, and MYREN. The directed loop corresponds to a bus transport network that provides loop service with $M_O=M_D=6$ origin and destination bus stops. In comparison to the topology of the networks of the last section which have the same number of origin and destination nodes, the directed loop with only 11 ADE performs worst. This results from a larger average overlapped index $\bar{\chi }=6.51$ due to a greater number of overlapped edges and parasitic origin nodes which implies poorer accuracy as it is harder to disentangle the various ODs. Nonetheless, the directed loop is the only network amongst those in Fig. 4a–d where all origin nodes display essentially equivalent performance. The indices $\chi _i$ for these origins are 6.00, 6.60, 6.91, 6.93, 6.63, 6.00, respectively, which are highly similar and consistent with them having comparable accuracies. The directed loop offers no alternative shortest path, so every agent from origin *i* to destination *j* takes the same path. The application of the directed loop for a bus loop service will be detailed in the next section.

For the internet networks VinaREN and MYREN, they have more destinations $M_D$ to deal with, and generally report larger errors in predicting the most popular destination. Their ADE are comparable to the smaller networks in Fig. 4b–d, such that they do not quite possess proportionately sufficient ADE to deal with more nodes. This is a consequence of the features that many real-world internet networks are scale-free and small-world^34,35^. Whilst these two networks are relatively small to be considered anywhere near being scale-free, this is nonetheless plausibly the situation with scale-free networks which are ultra small^36,37^: Scale-free networks probably have comparably poorer performance due to the ultra few ADE. In the case of VinaREN and MYREN, this small-world feature leads to the presence of common highway edges which cause considerable overlapping of edges. This explains the worst performance of VinaREN and MYREN compared to the rest of the network topologies. In addition, because of the presence of alternative shortest paths in the MYREN network relative to the VinaREN network, which introduces additional uncertainties, the MYREN network performs worse compared to VinaREN.

### Application: bus loop service

**Figure 11:**
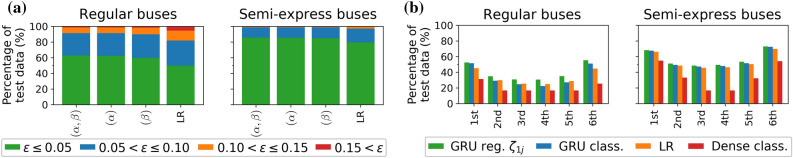
(**a**) Regression: predicting $\zeta _{1j}$ by DNNGRU using ($\alpha ,\beta$), $\alpha$, $\beta$; and by LR using $\alpha$. (**b**) Classification: predicting the most, second most, $\cdots$, least likely destinations from origin $i=1$ by three different DNN architectures, and LR using $\alpha$.

We consider a loop of $M_O$ origin bus stops with $M_D$ destination bus stops served by *N* buses in the form of Fig. 4a. Without loss of generality, origins are placed before destinations, all staggered. Regular buses go around the loop serving all bus stops sequentially. To describe other complex network topologies in a bus system, we additionally implement a *semi-express* configuration where buses only board commuters from a subset of origins whilst always allow alighting^29^. This configuration turns out to be chaotic, but outperforms regular or fully express buses in minimising commuters waiting time with optimal performance achieved at the edge of chaos^30^. Let $N=M_O=M_D=6$. This system represents a morning commute scenario where students living in the NTU campus residences commute to faculty buildings (Fig. 1d). (The map shows 7 residences and 5 faculty buildings. We approximate this with $M_O=M_D=6$). The complex network for semi-express buses (carriers, i.e. edges) are depicted in Fig. 1e. Each semi-express bus boards people from three origins, then allows alighting at every destination. Different semi-express buses do not board from the same origin subset.

Unlike edges in general complex networks, each bus in a bus network maintains its identity as a carrier between nodes. In other words, passengers from different buses do not mix even if the buses share the same road. On the other hand for general complex networks, an edge connecting a pair of nodes would combine all agents that traverse it. This makes bus systems relatively easier to be analytically treated, and so we can compare performances by DNNGRU and LR.

We can simulate each of these two bus systems given some $\zeta _{ij}$ to generate: $\alpha$) the number of commuters on the buses after leaving a bus stop; and $\beta$) the duration that the buses spend at bus stops. Our parameters are based on real data measured from NTU campus shuttle buses^26,27,38^. We record 10 laps of service for each bus. This is reasonable as a bus takes $\sim$ 20 minutes a loop corresponding to 3.5 h of service for the morning commute from 8 am to $11\text {:}30$ am. All buses’ time series are concatenated into one single feature, for each $\alpha$ and $\beta$. Empirically, this longer time series turns out to significantly improve performance, compared to each bus representing separate features. As before, DNN training comprises 250 k datasets, with another 10 k as validation during training. Additionally, 50 k datasets are for testing. The latter are also used by LR to predict $\zeta _{ij}$. The various DNN architectural details are given in Methods.

With respect to $i=1$ (other origins $i\ne 1$ to be trained separately), DNNGRU predicts $\zeta _{1j}$ using ($\alpha ,\beta$), $\alpha$, or $\beta$. LR predicts $\zeta _{ij}$ for all *i*, using $\alpha$ where $(x_i,y_j)$ are directly deducible, and from which we take $\zeta _{1j}$ for comparison. Figure 11a shows the performance of DNNGRU clearly outperforming LR in predicting $\zeta _{1j}$. The green, blue, orange, red bars respectively represent $\varepsilon \le 0.05$, $0.05<\varepsilon \le 0.10$, $0.10<\varepsilon \le 0.15$, $0.15<\varepsilon$, where $\varepsilon$ is the difference between the predicted $\zeta _{1j}$ and the true $\zeta _{1j}$. Generally, using $\alpha$, $\beta$ or both for DNNGRU are equally good. A semi-express network topology has higher prediction accuracy than that of regular buses since it allows for more diverse data to learn from. The former topology is also a more efficient bus system^29^, and conceivably in general complex networks as it provides greater variety of pathways than one single loop (as discussed in section “General complex networks”).

From $\zeta _{1j}$ obtained by DNNGRU and LR using $\alpha$, we order the destination ranking for origin $i=1$ according to their probabilities and display their prediction accuracies in Fig. 11b. These are compared with directly classifying destination ranking by GRU or dense layers using $\alpha$. Direct classification requires training individual DNNs for each rank, i.e. one DNN learns to predict the most likely destination, another learns to predict the second most likely one, etc. Despite having dedicated DNNGRUs to classify each rank, they turn out to always be inferior to one DNNGRU predicting $\zeta _{1j}$ to then obtain the ranking. Intriguingly, they are only marginally superior (but not always) to LR. Consequently, if there are insufficient training data for DNNGRU, LR serves as a quick estimate with respectable accuracy compared to DNNGRU. Dense DNNs are consistently poorest due to its simplicity in not inferring temporal information in the time series, and also nowhere comparable to LR. DNNGRU regressing $\zeta _{1j}$ is always the best. Architectural details on using DNNGRU for direct classification and dense DNN for classification are given in Methods.

Classifying destination ranking produces a *U*-shape, with predicting the least likely destination being most accurate. This is generally true for all the networks studied in Fig. 4 as well, when trying to predict the other rankings other than the most popular destination. Errors in predicting $\zeta _{1j}$ may mess up the ordering, and those in between (2nd most likely, $\cdots$, 2nd least likely) can be affected both above and below. The most and least likely ones are only affected from below or above, respectively, with the latter being bounded by zero—giving it slightly better accuracy. Incidentally, DNN direct classification generally tends to overfit from excessive training, with test accuracy systematically less than train accuracy. Conversely, DNN regressing for $\zeta _{1j}$ does not seem to suffer from overfitting with test accuracy remaining comparable to train accuracy.

These results are useful for practical applications, as it informs us which bus stops should be prioritised and which may be skipped if needed. Train loops are another common loop services. For instance, major cities in the world have light rail system connected to rapid transit system. Unlike buses, trains have dedicated tracks free from traffic. Therefore, they are cleanly scheduled to stop over prescribed durations and do not experience bunching. This implies $\beta$ is not applicable, as the duration a train spends at a station is not proportional to demand. Nevertheless, $\alpha$ is measurable for inferring the OD distribution $\zeta _{ij}$ of this train loop. Moreover, we can use DNN with supervised learning which is network-free on routes with multiple loops and linear/branching topologies.

## Discussion

The main purpose of our paper is to introduce a machine learning approach to infer origin-destination (OD) distribution in a complex network. More specifically, we implement a recurrent deep neural network architecture, which is trained by supervised learning using labelled data. These labelled data comprise the number of agents at every time step on each link/edge of the network, with the labels being the OD probability distribution.

The motivation for employing a recurrent neural network (as opposed to other non-neural network machine learning approach) is that it allows for processing temporal information present within the time-series input data on the number of agents on each link/edge of the network. Furthermore, when using a recurrent neural network, the same architecture can be used regardless of the time-series length. We tried both LSTM and GRU architectures and found the accuracy results to be essentially identical, with the latter requiring significantly less computational resources. This is why we focused on DNNGRU and demonstrated its application throughout this paper.

Whilst there have been extensive methods in the literature of OD inference, we believe that the performance of our DNNGRU is fairly evaluated by comparison with analytical solutions of the expected values, especially in some small networks where such analytical solutions of the expected values can be computed. Therefore, we calculated such expected values using linear regression in the case of the loop network and the small lattice network for comparison with the results from DNNGRU. Additionally, we compared our results with some other methods like expectation-maximisation, Bayesian inference and entropy minimisation. We find that providing analytical solution for the expected value and comparison with this should provide sufficient demonstration of our DNNGRU results, as opposed to directly evaluating the vast other methods found in the literature.

Our DNNGRU does not require knowledge of the underlying topology of the network. We only need to track the number of agents on each link/edge of the network. Hence, we do not need to provide the adjacency matrix which are required in most other approaches like Vardi’s expectation-maximisation, Bayesian inference, and even graph neural networks (GNN). This is a key advantage of our supervised learning approach using DNNGRU instead of GNN.

### Scalability

Scalability is always an issue with any algorithm/method. For small networks that we investigated here, we used “most popular destination” as one of our metrics. With many more nodes in a larger network, our DNNGRU can just increase the number of nodes in the input and output layers accordingly. This is demonstrated by our implementation on VinaREN and MYREN which are larger than 6 origins and 6 destinations. For a recurrent neural network (like our DNNGRU), the computational time is dictated primarily by the length of the time-series input, not the number of nodes within the input and output layers. This is because the latter can be computed in parallel but the former must be computed sequentially.

In terms of memory usage, larger network with more nodes would contain more edges. Since our input data comprises number of agents on edges, then the memory would scale proportionally with the number of edges. As most real-world networks tend to be scale-free and/or small-world, the number of edges grow much slower than the number of nodes in the network. This is illustrated in our two real-world network examples of VinaREN and MYREN (see Fig. 4): They have 25 and 37 nodes respectively as compared to 12 nodes in our smaller networks (loop, lattice, random, small world). However, the VinaREN and MYREN networks only have ADE (active directed edges) of 24 and 40, respectively, which are comparable to the ADEs of our smaller networks (11, 38, 32, 24, respectively). In other words, these examples show that whilst the number of nodes are $2\times$ or $3\times$ more, the number of ADE remains of the same order. Hence, the memory requirement for larger networks remains modest.

However, the accuracy of trying to predict the single most popular destination when scaling up the number of nodes would inevitably suffer. This is true for any algorithm or model trying to make such a singular prediction. Perhaps with more nodes like 1000 destinations, or 1,000,000, instead of asking for the single most popular destination (which would certainly be difficult), we can ask for the “top 10% popular destination”. This provides a way of scaling with the number of nodes to obtain meaningful results.

### Concluding remarks

Knowledge of $\zeta _{ij}$ is akin to possessing knowledge of the dynamical law of the system. With respect to this dynamical law, we can figure out optimal configurations of buses for delivery of commuters from their origins to desired destinations. As a concrete implementation, the latter is achieved via multi-agent reinforcement learning (MARL) recently carried out^29^. However, arbitrary $\zeta _{ij}$ were tested there like uniform distribution (fixed proportion of commuters alighting at every destination) or antipodal (commuters alight at the destination opposite where they boarded on the loop). More complicated $\zeta _{ij}$ can certainly be prescribed for the MARL framework in Refs.^29,39^, but the most useful one would be the actual $\zeta _{ij}$ corresponding to the bus loop service being studied, like our NTU campus loop shuttle bus service^27,28,38^. Thus, the algorithm presented here is complementary to the MARL framework in Ref.^29^. We intend to further implement our algorithm to city-wide bus networks from publicly accessible data^26^ with MARL optimisation, to be reported elsewhere. Other complex systems (c.f. Fig. 1) can be similarly modelled towards improving the efficiency of agent-transfer or impeding undesirable transactions.

In studying general complex networks, we revealed how different network topologies can lead to (dis)advantageous performances, quantifying in terms of $\chi _i$ and the presence of alternative shortest paths. We also studied how different origin nodes’ properties may make them perform slightly better than other origin nodes, in the examples of the lattice, random, small-world networks, as well as real-world networks like the loop, VinaREN and MYREN. Notably, we demonstrated empirically that the use of recurrent neural network architecture like GRU allows for longer time-series data to generally yield more accurate predictions, with the time-series data length and prediction error appearing to scale as a power law.

Whilst we have illustrated a concrete application in a university bus loop service, the framework presented here can be used in various other areas like Internet tomography^1,3,7,10,40^, city-wide bus/traffic networks^3–5,12,14,15,17,35,41–53^, as well as in mapping global epidemic/pandemic propagation and contact tracing^16,18,22,54–62^. As we have shown how DNNGRU and our formulation of linear regression consistently outperform existing methods using expectation-maximisation with moments^7^, Bayesian inference^3^ and entropy maximisation^10^, we expect further impactful advancements in OD inference based on this work. With DNNGRU being network-free where it only requires training it with data by supervised learning with no requirement of analytical modelling, this approach is scalable. We envision significant improvements in OD mapping in these other fields, bringing with them major enhancements whilst respecting privacy.

## Methods

### DNNGRU hyperparameters

We employ a deep neural network (DNN) with two hidden layers consisting of gated recurrent units (GRU). The input layer comprises the measurement of the number of agents on each active directed edge (ADE) of a complex network at some moment in time. If there are *L* ADE, then there are *L* units in the input layer. As the next two (hidden) layers are GRUs stacked on one another, the input data to the input layer can contain *T* time steps. In other words, each of the *L* units in the input layer comprises a time series of length *T*. Finally, the output layer comprises $M_D$ units (recall that $M_D$ is the number of destination nodes), each giving the probability of agents from some specific origin node *i* to end up at destination *j*. Different DNNs are trained individually for different origin nodes *i*. (See Fig. 2).

For a given setup, the input layer and output layer have fixed number of units. So, the number of GRU hidden layers and the number of units in each GRU hidden layer are user-defined. We fix each GRU hidden layer to have 128 units. We observe that increasing the width of the hidden layers generally improve performance. However, the number of trainable weights and biases in the DNNGRU would rapidly blow up with increasing width size. The choice of 128 units for each hidden GRU layer balances this, with $\sim$ 150 k trainable weights and biases, which can be well-trained by 250k training datasets. On the other hand, whilst two hidden layers definitely outperform one hidden layer, three or more hidden layers do not show improved performance over two hidden layers. Thus, two hidden layers seem optimal.

DNNGRU training is carried out on TensorFlow 2.3 using Keras^63^. Default activation function is used for GRU, whilst the output layer uses sigmoid. Although the sum of all output values is 1 (c.f. the summation equation below Eq. (1)), softmax does not seem desirable as it sometimes leads to stagnant training. The loss function used is binary cross entropy, optimised with the standard ADAM. An $L^2$ recurrent regularisation is applied on the GRU hidden layers. This prevents blowing up of the weights which occasionally occurs during training if no regularisation is used. Batch size of 128 is used, which is generally better than 64 or 32. However, larger batch sizes would tax the GPU VRAM, leading to sporadic GPU breakdowns. Training is carried out over 200 epochs. Generally, optimal performance is achieved well before 200 epochs and continues to incrementally improve. No significant overfitting is observed, so no early stopping is implemented. In fact, for many of the DNNs, training accuracy is essentially similar to validation accuracy. Sometimes, the latter is slightly less than the former, but typically continues to improve over training epochs. Incidentally, input quantities which are small ($\ll 100$) are left as they are, whilst larger input values ($\gtrsim 100$) are better to be rescaled by appropriate division, so that the DNN performs optimally.

### Transfer learning

With GRU layers, the input layer can take any time series length *T* and as long as the number of ADE *L* remains the same, then the DNNGRU architecture remains the same. A longer *T* would require longer training time since the time series data are processed sequentially by the DNNGRU and cannot be parallelised. This unmodified DNNGRU architecture regardless of *T* allows the implementation of transfer learning when supplying inputs of different temporal lengths *T* to obtain each plot point in Figs.  3, 5, 6, 7. So with respect to each origin node *i*, a DNNGRU is trained completely from scratch with $T=1000$. After this has completed, the trained weights are used as initial weights for the next shorter time series with $T=750$, and trained for only 20 epochs instead of the full 200 epochs. This generally leads to optimised performance similar to that from complete training with random initial weights (we carried out some tests and this is generally true), but only takes $10\%$ of training time. Then, the trained weights for $T=750$ are used as initial weights to train a DNNGRU for $T=500$, and so on until $T=1$.

### The coefficient of determination $r^2$

The coefficient of determination, $r^2$ is given by

3 $$\begin{aligned} r^2&=1-\frac{\sum {\left( \zeta _{ij, predicted}-\zeta _{ij, true}\right) ^2}}{\sum {\left( \zeta _{ij, predicted}-\overline{\zeta _{ij, predicted}}\right) ^2}}, \end{aligned}$$

where $\overline{\zeta _{ij, predicted}}$ is the mean of all $\zeta _{ij, predicted}$. This imposes an exact fit of $\zeta _{ij, predicted}=\zeta _{ij, true}$ with zero intercept, as opposed to the correlation coefficient of a linear regression fit that allows for an unspecified intercept to be determined from the fitting. Note that the coefficient of determination $r^2$ can take negative values, which would imply that the predictions are worse than the baseline model that always predicts the mean $\overline{\zeta _{ij, predicted}}$ (i.e. $\zeta _{ij, true}=\overline{\zeta _{ij, predicted}}$, giving $r^2=0$). Hence, $r^2<0$ are not shown in the plots in Figs. 3, 5, 6, 7, as they imply that the predictions are just so poor, they are essentially nonsensical. In contrast, $r^2=1$ implies perfect predictions (since *every* $\zeta _{ij, predicted}=\zeta _{ij, true}$).

### Vardi’s expectation-maximisation algorithm

We present an analytical comparison between linear regression (LR) and Vardi’s expectation-maximisation (EM), to elucidate how a switch in paradigm from trying to infer actual whole numbers of the OD matrix to instead inferring the probabilities would lead to significant improvements in the predictions. It is instructive to consider a directed loop for this purpose as shown in Fig. 4a, which is also what happens when a bus picks up people from $M_O$ origin bus stops and then delivers them at $M_D$ destination bus stops (c.f. Fig. 1d). This bus system is the subject of section “Application: bus loop service”, which is based on the NTU shuttle bus service^27^.

The bus would pick up $x_i$ commuters from each of the origin bus stops ($i=1,\ldots ,M_O$), and then let $y_j$ commuters alight at each of the destination bus stops ($j=1,\ldots ,M_D$) according to $\zeta _{ij}$, which leads to Eq. (1). In other words, everybody who alights at destination bus stop *j* for each $j=1,\ldots ,M_D$ comprises everybody who boarded from each of the origin bus stops who wants to go to bus stop *j*. Since we can repeatedly measure all the $x_i$ and $y_j$ as the bus loops around over time, Eq. (1) is naturally in a form where LR is directly applicable. The OD probabilities $\zeta _{ij}$ are coefficients of a multivariate system of linear equations, and the best set of values of $\zeta _{ij}$ is the one that would minimise the mean squared error of the predicted $y_j$ given the $x_i$ according to Eq. (1), versus the measured $y_j$.

On the other hand, Vardi^7^ approaches the problem differently. Instead of evaluating the OD number of commuters from the OD probabilities $\zeta _{ij}$, Vardi determines the actual OD whole numbers of commuters by modelling them directly as Poisson random variables with mean $\lambda _k$, where $k=1,\ldots ,M_OM_D$. With the correct Poisson parameters $\lambda _k$ representing the mean (and variance) of each OD from some $O_i$ to some $D_j$, the actual number of people who want to go from $O_i$ to $D_j$ is the random variable $X_k\sim \text {Poisson}(\lambda _k)$.

Without loss of generality, let us fix $M_O=M_D=6$ as in Fig. 4a. This gives the following matrix equation:

4 $$\begin{aligned} \vec {Y}&=A\vec {X}. \end{aligned}$$

The column vector $\vec {X}$ is the collection of all 36 possible OD for this network. More specifically, we define

5 $$\begin{aligned} \vec {X}= \left( X_{O_1D_1},X_{O_1D_2},X_{O_1D_3},X_{O_1D_4},X_{O_1D_5},X_{O_1D_6},X_{O_2D_1},X_{O_2D_2},\ldots ,X_{O_6D_6}\right) ^T, \end{aligned}$$

with $X_{O_iD_j}$ denoting the actual number of people who go from $O_i$ to $D_j$. The column vector $\vec {Y}$ comprises the number of commuters on each of the ADE, i.e. the 11 edges that are being used in this loop: $O_1O_2,O_2O_3,\ldots ,D_5D_6$. Explicitly,

6 $$\begin{aligned} \vec {Y}=\left( Y_{O_1O_2}, Y_{O_2O_3},\ldots ,Y_{D_5D_6}\right) ^T, \end{aligned}$$

where $Y_{O_1O_2}$ is the number of people on the directed edge $O_1O_2$, etc. Then, *A* is an $11\times 36$ matrix that relates the vector $\vec {X}$ of number of people for each OD to the vector $\vec {Y}$ of number of people on each ADE. This matrix *A* is referred to as the “routing matrix”. For this loop network, we can determine *A* to be:

7 $$\begin{aligned} A=\left( \begin{array}{cccccccccccccccccccccccccccccccccccc} 1 &{} 1 &{} 1 &{} 1 &{} 1 &{} 1 &{} 0 &{} 0 &{} 0 &{} 0 &{} 0 &{} 0 &{} 0 &{} 0 &{} 0 &{} 0 &{} 0 &{} 0 &{} 0 &{} 0 &{} 0 &{} 0 &{} 0 &{} 0 &{} 0 &{} 0 &{} 0 &{} 0 &{} 0 &{} 0 &{} 0 &{} 0 &{} 0 &{} 0 &{} 0 &{} 0\\ 1 &{} 1 &{} 1 &{} 1 &{} 1 &{} 1 &{} 1 &{} 1 &{} 1 &{} 1 &{} 1 &{} 1 &{} 0 &{} 0 &{} 0 &{} 0 &{} 0 &{} 0 &{} 0 &{} 0 &{} 0 &{} 0 &{} 0 &{} 0 &{} 0 &{} 0 &{} 0 &{} 0 &{} 0 &{} 0 &{} 0 &{} 0 &{} 0 &{} 0 &{} 0 &{} 0\\ 1 &{} 1 &{} 1 &{} 1 &{} 1 &{} 1 &{} 1 &{} 1 &{} 1 &{} 1 &{} 1 &{} 1 &{} 1 &{} 1 &{} 1 &{} 1 &{} 1 &{} 1 &{} 0 &{} 0 &{} 0 &{} 0 &{} 0 &{} 0 &{} 0 &{} 0 &{} 0 &{} 0 &{} 0 &{} 0 &{} 0 &{} 0 &{} 0 &{} 0 &{} 0 &{} 0\\ 1 &{} 1 &{} 1 &{} 1 &{} 1 &{} 1 &{} 1 &{} 1 &{} 1 &{} 1 &{} 1 &{} 1 &{} 1 &{} 1 &{} 1 &{} 1 &{} 1 &{} 1 &{} 1 &{} 1 &{} 1 &{} 1 &{} 1 &{} 1 &{} 0 &{} 0 &{} 0 &{} 0 &{} 0 &{} 0 &{} 0 &{} 0 &{} 0 &{} 0 &{} 0 &{} 0\\ 1 &{} 1 &{} 1 &{} 1 &{} 1 &{} 1 &{} 1 &{} 1 &{} 1 &{} 1 &{} 1 &{} 1 &{} 1 &{} 1 &{} 1 &{} 1 &{} 1 &{} 1 &{} 1 &{} 1 &{} 1 &{} 1 &{} 1 &{} 1 &{} 1 &{} 1 &{} 1 &{} 1 &{} 1 &{} 1 &{} 0 &{} 0 &{} 0 &{} 0 &{} 0 &{} 0\\ 1 &{} 1 &{} 1 &{} 1 &{} 1 &{} 1 &{} 1 &{} 1 &{} 1 &{} 1 &{} 1 &{} 1 &{} 1 &{} 1 &{} 1 &{} 1 &{} 1 &{} 1 &{} 1 &{} 1 &{} 1 &{} 1 &{} 1 &{} 1 &{} 1 &{} 1 &{} 1 &{} 1 &{} 1 &{} 1 &{} 1 &{} 1 &{} 1 &{} 1 &{} 1 &{} 1\\ 0 &{} 1 &{} 1 &{} 1 &{} 1 &{} 1 &{} 0 &{} 1 &{} 1 &{} 1 &{} 1 &{} 1 &{} 0 &{} 1 &{} 1 &{} 1 &{} 1 &{} 1 &{} 0 &{} 1 &{} 1 &{} 1 &{} 1 &{} 1 &{} 0 &{} 1 &{} 1 &{} 1 &{} 1 &{} 1 &{} 0 &{} 1 &{} 1 &{} 1 &{} 1 &{} 1\\ 0 &{} 0 &{} 1 &{} 1 &{} 1 &{} 1 &{} 0 &{} 0 &{} 1 &{} 1 &{} 1 &{} 1 &{} 0 &{} 0 &{} 1 &{} 1 &{} 1 &{} 1 &{} 0 &{} 0 &{} 1 &{} 1 &{} 1 &{} 1 &{} 0 &{} 0 &{} 1 &{} 1 &{} 1 &{} 1 &{} 0 &{} 0 &{} 1 &{} 1 &{} 1 &{} 1\\ 0 &{} 0 &{} 0 &{} 1 &{} 1 &{} 1 &{} 0 &{} 0 &{} 0 &{} 1 &{} 1 &{} 1 &{} 0 &{} 0 &{} 0 &{} 1 &{} 1 &{} 1 &{} 0 &{} 0 &{} 0 &{} 1 &{} 1 &{} 1 &{} 0 &{} 0 &{} 0 &{} 1 &{} 1 &{} 1 &{} 0 &{} 0 &{} 0 &{} 1 &{} 1 &{} 1\\ 0 &{} 0 &{} 0 &{} 0 &{} 1 &{} 1 &{} 0 &{} 0 &{} 0 &{} 0 &{} 1 &{} 1 &{} 0 &{} 0 &{} 0 &{} 0 &{} 1 &{} 1 &{} 0 &{} 0 &{} 0 &{} 0 &{} 1 &{} 1 &{} 0 &{} 0 &{} 0 &{} 0 &{} 1 &{} 1 &{} 0 &{} 0 &{} 0 &{} 0 &{} 1 &{} 1\\ 0 &{} 0 &{} 0 &{} 0 &{} 0 &{} 1 &{} 0 &{} 0 &{} 0 &{} 0 &{} 0 &{} 1 &{} 0 &{} 0 &{} 0 &{} 0 &{} 0 &{} 1 &{} 0 &{} 0 &{} 0 &{} 0 &{} 0 &{} 1 &{} 0 &{} 0 &{} 0 &{} 0 &{} 0 &{} 1 &{} 0 &{} 0 &{} 0 &{} 0 &{} 0 &{} 1 \end{array}\right) . \end{aligned}$$

For example, the first component in Eq. (4) says that everybody picked up from $O_1$ must go via $O_1O_2$, regardless of whether they are going to $D_1,\ldots ,D_6$. So $Y_1=\text {"total number of people on the directed edge } O_1O_2''$ is the sum of all the people originating from $O_1$. The second component says that everybody picked up from $O_1$ and $O_2$ must go via $O_2O_3$, etc., until the sixth component, which is the sum of every commuter since they must all pass via $O_6D_1$. Now the 7th component refers to the number of commuters on the directed edge $D_1D_2$. Since everybody who wants to go to $D_1$ has alighted, they do not traverse this edge regardless of which origin they came from. So $Y_7$ excludes $X_{O_1D_1},X_{O_2D_1},X_{O_3D_1},X_{O_4D_1},X_{O_5D_1},X_{O_6D_1}$, and so on for the rest of the components of $\vec {Y}$. This is how the routing matrix *A* is constructed.

It is obvious in Eq. (4) that there are 36 unknown components of $\vec {\lambda }=(\lambda _1,\ldots ,\lambda _{36})^T$, with $\vec {X}\sim \text {Poisson}(\vec {\lambda })$. However, there are only 11 independent equations, corresponding to each of the rows in Eq. (4). This is typical of OD inference problems where the number of ADE that provide measurements is less than the number of OD. Vardi’s approach is to figure out the maximum likelihood of $\vec {\lambda }$ from observing $\vec {Y}$. The key idea of overcoming the problem of underdetermination is to make use of moments (as well as the normal approximation given a large sample of measured $\vec {Y}$’s) to generate more and sufficient equations (details in Ref.^7^), leading to an iterative algorithm that would converge to the optimal $\vec {\lambda }$. Let $\bar{\vec {Y}}$ be the mean of all $\vec {Y}$’s which are sampled by measuring the number of commuters at each ADE, and $\vec {S}$ be the corresponding covariance matrix arranged as a column vector. On top of that, let *B* be a matrix obtained from *A* where each row of *B* is the element-wise product of a pair of rows of *A* (details in Ref.^7^). Note that $\vec {S}$ and *B* are the result of the normal approximation and the use of first and second order moments to generate more equations. Vardi then constructs the following augmented matrix equation

$$\begin{aligned} \begin{pmatrix}\bar{\vec {Y}}\\ \vec {S}\end{pmatrix}= \begin{pmatrix}A\\ B\end{pmatrix}\vec {\lambda }. \end{aligned}$$

This results in an iterative algorithm for computing $\vec {\lambda }$:

8 $$\begin{aligned} \lambda _k&\leftarrow \frac{\lambda _k}{\sum _i{a_{ik}}+\sum _i{b_{ik}}}\nonumber \\&\times \left( \sum _i{\frac{a_{ik}\bar{Y}_i}{\sum _p{a_{ip}\lambda _p}}}+\sum _i{\frac{b_{ik}S_i}{\sum _p{b_{ip}\lambda _p}}}\right) . \end{aligned}$$

Here, $\lambda _k,\bar{Y}_i,S_i,a_{ik},b_{ik}$ are the components of $\vec {\lambda },\bar{\vec {Y}},\vec {S},A,B$, respectively. Once $\vec {\lambda }$ has been found, then we can normalise with respect to each origin to obtain the probabilities $\zeta _{ij}$.

### DNN architecture for bus loop service

For the bus loop service, there are *N* buses serving the loop of bus stops. Unlike the situation for the general complex networks, here the input data comprises number of people on the bus after the bus leaves a bus stop, $\alpha$; and/or the duration the bus spends stopping at a bus stop, $\beta$. In principle, we can let each input unit be the number of people on each bus, and/or the duration the bus spends stopping at a bus stop as a second feature for the input. Nevertheless, we find that concatenating these time series from all buses into one single long time series as the input turns out to empirically boost performance. This perhaps arises due to complex interactions amongst the buses, viz. the number of people picked up by a leading bus affects the remaining number of people picked up by trailing buses.

Hence for DNNGRU, the input layer comprises $\alpha$ and/or $\beta$, where these features are the concatenation of the time-series of all *N* buses. If only $\alpha$ or $\beta$ is used, then the input layer comprises that one single unit with a concatenated time-series. The rest is the same as the DNNGRU architecture used for general complex networks, viz. two hidden GRU layers with default activation each with width of 128 units, followed by an output layer with $M_D$ units with a sigmoid activation. So the output is $\zeta _{ij}$ for each destination *j*.

For directly classifying the rank of destination bus stop, the loss function used is sparse categorical cross entropy, which is the standard loss function used in a classification problem with many labels. Then, an argmax is applied to the output layer, such that the unit with the largest value is deemed as the prediction of the destination bus stop of the stipulated rank. This loss function compares the correctness of the predicted destination bus stop with respect to the actual destination bus stop. Direct classification differs from directly regressing for the values of $\zeta _{ij}$ where the loss function used is binary cross entropy that measures the closeness of the values of the predicted $\zeta _{ij}$ from the true value. Finally in the case where dense layers are used in the direct classification, the GRU units are simply replaced by regular densely connected feedforward layers. The input layer is not one single feature, but comprises all values of $\alpha$ which is then fed into two densely connected layers each of width 128 units followed by an output layer with $M_D$ units.

## Supplementary Information

Supplementary Information.

## Data Availability

The data that support the findings of this study are available from the corresponding author upon reasonable request.
