# Supplementary material for: Inferring origin-destination distribution of agent transfer in a complex network using deep gated recurrent units
Source: Sci Rep. 2023 May 22;13:8287. doi: 10.1038/s41598-023-35417-9 (PMC10202365; doi:10.1038/s41598-023-35417-9)
Supplement: Supplementary file 1 — Supplementary Information. [file 41598_2023_35417_MOESM1_ESM.pdf]

Inferring origin-destination distribution of agent transfer in a complex network using deep gated recurrent units - Supplemental material

Vee-Liem Saw[1], Luca Vismara[2], Suryadi[3], Bo Yang[4], Mikael Johansson[5], and Lock Yue Chew[6]

[1]Vee-Liem@ntu.edu.sg
[2]vism0001@e.ntu.edu.sg
[3]sury0013@e.ntu.edu.sg
[4]yang.bo@ntu.edu.sg
[5]mikaelj@kth.se
[6]lockyue@ntu.edu.sg
[1,2,3,4,6]Division of Physics and Applied Physics, School of Physical and Mathematical Sciences, Nanyang Technological University, Singapore
[5]School of Electrical Engineering, KTH Royal Institute of Technology, Stockholm, Sweden

ABSTRACT

This is the supplemental material to the main text.

1 Linear regression for Vardi's network

Vardi's network[1] is defined by the routing matrix A, given by:

$$A = \begin{pmatrix} 1 & 0 & 0 & 0 & 0 & 0 & 0 & 0 & 0 & 0 & 0 & 0 \\ 0 & 1 & 1 & 0 & 0 & 1 & 0 & 0 & 0 & 0 & 0 & 0 \\ 0 & 0 & 0 & 1 & 0 & 1 & 1 & 0 & 0 & 1 & 0 & 0 \\ 0 & 0 & 0 & 0 & 1 & 0 & 0 & 0 & 0 & 0 & 0 & 0 \\ 0 & 0 & 0 & 0 & 0 & 0 & 1 & 1 & 0 & 1 & 1 & 0 \\ 0 & 0 & 1 & 0 & 0 & 1 & 0 & 0 & 1 & 0 & 0 & 0 \\ 0 & 0 & 0 & 0 & 0 & 0 & 0 & 0 & 0 & 1 & 1 & 1 \end{pmatrix}. \tag{1}$$

This routing matrix relates the actual number of agents contained in the 12 OD components of $\vec{X}$, and the number of agents at each of the 7 edges in his network $\vec{Y}$, i.e.

$$\vec{Y} = A\vec{X}. \tag{2}$$

The 12 components of $\vec{X}$ comprise the number of agents going from a to b, a to c, a to d, b to a, b to c, b to d, c to a, c to b, c to d, d to a, d to b, d to c. The 7 components of $\vec{Y}$ comprise the number of agents on the directed edges ab, ac, ba, bc, cb, cd, dc. We denote their components X_{ab} as the number of agents from origin a to destination b, and Y_{ab} as the number of agents on the directed edge ab, etc. (See[1], as these definitions are taken directly from his network given there.)

Unlike our networks where we consider agents propagating in time through the edges sequentially, Vardi views the data as binned over a time interval. In other words, he assumes that over this time interval, if given $\vec{X}$ of all the 12 possible OD, then the number of agents $\vec{Y}$ on the 7 edges over this time interval are given by Eq. (2). This view is in fact also explicitly stated in a Bayesian approach that uses Vardi's network in Ref.[2] (see the abstract of that paper). For an observer, we are only given information of $\vec{Y}$, and the task is to infer the $\vec{X}$ that gave rise to the observation. As there are only 7 equations for 12 unknowns in Eq. (2), this is an underdetermined system.

Vardi's EM algorithm has been elaborated in the Methods section of the main text. Here, we provide the solution by linear regression of the probabilities ζ_{ij}, which is possible if there are additional data: the total number of agents originating from each node, $\vec{V} = (V_a, V_b, V_c, V_d)^T$. This is reasonably obtainable as a counter can track how many agents are leaving each origin

node without knowing where they want to go. In the case of a bus system, this is simply tracking how many people are boarding the bus at each bus stop.

With knowledge of $\vec{V}$, we can write $\vec{X}$ in terms of $\vec{V}$ and the probabilities ζ_{ij}. These probabilities ζ_{ij} are unknown, and now become the variables of interest. From a set of data samples of $\vec{X}$, these cannot be combined as different samples have different sets of OD. Nevertheless under the assumption that the probabilities are constant during the period of interest, the samples can now be combined to regress for this common ζ_{ij}. Eq. (2) becomes:

$$Y_{ab} = \zeta_{ab}V_a \tag{3}$$
$$Y_{ac} = (1-\zeta_{ab})V_a + \zeta_{bd}V_b \tag{4}$$
$$Y_{ba} = (1-\zeta_{bc})V_b + \zeta_{ca}V_c + \zeta_{da}V_d \tag{5}$$
$$Y_{bc} = \zeta_{bc}V_b \tag{6}$$
$$Y_{cb} = (1-\zeta_{cd})V_c + (1-\zeta_{dc})V_d \tag{7}$$
$$Y_{cd} = \zeta_{ad}V_a + \zeta_{bd}V_b + \zeta_{cd}V_c \tag{8}$$
$$Y_{dc} = V_d. \tag{9}$$

We have used the fact that probabilities originating from the same origin must go to one of the destinations, so that they all sum up to 1. Consequently, instead of 12 components of ζ_{ij}, we only have 8 independent components, viz. $\zeta_{ab}, \zeta_{ad}, \zeta_{bc}, \zeta_{bd}, \zeta_{ca}, \zeta_{cd}, \zeta_{da}, \zeta_{dc}$. Note also that the 7th equation $Y_{dc} = V_d$ is not useful, so we only have 6 independent equations.

Given measured data of $\vec{V}, \vec{Y}$, these 6 equations can be analytically solved for the 8 independent ζ_{ij} so that the predicted $\hat{\vec{Y}}$ as compared to the actual $\vec{Y}$ would minimise the mean squared error, analogous to Eq. (20). We summarise the analytical solution for these optimal ζ_{ij} as follows. Let σ be

$$\sigma = \begin{pmatrix}
2\sum_m V_a^2 & 0 & 0 & -\sum_m V_aV_b & 0 & 0 & 0 & 0 \\
0 & \sum_m V_a^2 & 0 & \sum_m V_aV_b & 0 & \sum_m V_aV_c & 0 & 0 \\
0 & 0 & 2\sum_m V_b^2 & 0 & -\sum_m V_bV_c & 0 & -\sum_m V_bV_d & 0 \\
-\sum_m V_aV_b & \sum_m V_aV_b & 0 & 2\sum_m V_b^2 & 0 & \sum_m V_bV_c & 0 & 0 \\
0 & 0 & -\sum_m V_bV_c & 0 & \sum_m V_c^2 & 0 & \sum_m V_cV_d & 0 \\
0 & \sum_m V_aV_c & 0 & \sum_m V_bV_c & 0 & 2\sum_m V_c^2 & 0 & \sum_m V_cV_d \\
0 & 0 & -\sum_m V_bV_d & 0 & \sum_m V_cV_d & 0 & \sum_m V_d^2 & 0 \\
0 & 0 & 0 & 0 & 0 & \sum_m V_cV_d & 0 & \sum_m V_d^2
\end{pmatrix}. \tag{10}$$

The solution is:

$$\begin{pmatrix} \zeta_{ab} \\ \zeta_{ad} \\ \zeta_{bc} \\ \zeta_{bd} \\ \zeta_{ca} \\ \zeta_{cd} \\ \zeta_{da} \\ \zeta_{dc} \end{pmatrix} = \sigma^{-1} \cdot \begin{pmatrix}
\sum_m V_a(V_a + Y_{ab} - Y_{ac}) \\
\sum_m V_aY_{cd} \\
\sum_m V_b(V_b - Y_{ba} + Y_{bc}) \\
\sum_m V_b(-V_a + Y_{ac} + Y_{cd}) \\
\sum_m V_c(-V_b + Y_{ba}) \\
\sum_m V_c(V_c + V_d - Y_{cb} + Y_{cd}) \\
\sum_m V_d(-V_b + Y_{ba}) \\
\sum_m V_d(V_c + V_d - Y_{cb})
\end{pmatrix}. \tag{11}$$

2 Other methods of comparison for Vardi's network: Entropy maximisation and Bayesian inference

We implement Willumsen's entropy maximisation method to infer the OD trip matrix for Vardi's network[3]. The method is a direct application of the formula given in Ref.[3] for entropy maximisation. This turns out to return poor results with coefficient

of determination peaking around $r^2 \sim 0.039$. Clearly, the assumption that the most likely trip matrix would correspond to the one with largest entropy or equivalently, one which minimises the information in the observed data is not necessarily the case with the way agents behave in general networks.

We also carry out a Bayesian inference of OD, whereby given a uniform prior of the parameter $\vec{\lambda}$ and the Poisson likelihood of observing $\vec{Y}$ given $\vec{\lambda}$, we can use Bayes's theorem to obtain the posterior distribution of the parameter $\vec{\lambda}$ given $\vec{Y}$. There exists several Bayesian inference methods which differ in some technical details in the literature[2,4,5]. For instance, Ref.[2] implemented their Bayesian inference on Vardi's original network in a manner that involves $\vec{X}$, but did not compare with Vardi's EM in terms of performance. Vardi himself also pointed out that their formulation of Bayesian inference is only applicable with one observation of $\vec{Y}$ and not directly generalisable to many samples of $\vec{Y}$. Nevertheless, Vardi did suggest a more direct way of implementing a Bayesian inference (without the need to involve $\vec{X}$) to obtain the posterior distribution of the parameter $\vec{\lambda}$ given $\vec{Y}$, which we adopt here. The details of our implementation are presented in Section 3 below. Here, we summarise the results of such a Bayesian inference applied to Vardi's network. It turns out that the model fits the data very poorly: the r^2 value is ~ -0.2, regardless of the amount of samples of $\vec{Y}$ that are given. Inspection of the fitting of the Bayesian model indicates that underdetermination (there are only 7 equations for 12 unknowns) leads to high uncertainty in the inference. The Bayesian fitting produces a joint posterior distribution for all these 12 unknown components of $\vec{\lambda}$, with rather high degree of spread around the mean values. Hence, the Bayesian model is saying that its posterior prediction of the mean value of $\vec{\lambda}$ is plagued by large uncertainty.

Recall that Vardi's EM creates more equations using moments, to overcome the underdetermination of the equations, whereas our LR introduced in this paper strives to regress for the common coefficients ζ_{ij} such that all samples of measurements can be combined. Instead, Bayesian inference and entropy maximisation methods do not address the underdetermination problem, and consequently are still burdened by the high uncertainty of their results.

3 Bayesian inference for Vardi's network

To implement a Bayesian inference on Vardi's network, we strive to figure out the probability of the Poisson parameters $\vec{\lambda}$ given the observation of m samples of $\vec{Y}^{(1)},\cdots,\vec{Y}^{(m)}$, i.e. $P(\vec{\lambda}|\vec{Y}^{(1)},\cdots,\vec{Y}^{(m)})$. Using Bayes's theorem, we have

$$P(\vec{\lambda}|\vec{Y}^{(1)},\cdots,\vec{Y}^{(m)}) = \frac{P(\vec{Y}^{(1)},\cdots,\vec{Y}^{(m)}|\vec{\lambda})P(\vec{\lambda})}{P(\vec{Y}^{(1)},\cdots,\vec{Y}^{(m)})}. \tag{12}$$

Here, $P(\vec{\lambda}) = \prod_{k=1}^{12} P(\lambda_k)$ is the prior for the parameters $\vec{\lambda}$ whose 12 components are the 12 OD Poisson parameters in Vardi's network. Well, recall that in Vardi's network, $\vec{Y} = A\vec{X}$, with A given by Eq. (1). Also, each component of $\vec{X}$ is a Poisson random variable with corresponding Poisson parameter $\vec{\lambda}$. Thus, each of the 7 components of $\vec{Y}$ is a sum of Poisson random variables, which is just a Poisson random variable with the corresponding sum of the Poisson parameters. This allows a direct fitting of the Bayesian model given the observations of $\vec{Y}$. The model fitting is done using the *pymc3* Python library. In general, such computation is significantly lengthier than Vardi's EM or the training of DNNGRU. As a comparison, training of DNNGRU using 250k datasets takes about 9 hours on an RTX2070 Super GPU, with testing on 50k datasets done within a minute. Vardi's EM takes two days on a CPU to test the same 50k datasets. But the Bayesian fit takes 3 days on a CPU to test merely 1k datasets!

4 Comparison amongst DNNGRU, LR, EM for the loop

Figs. 3(g, h, i) in the main text compares the performances amongst DNNGRU, analytically solving the linear regression of data points with respect to Eq. (13), and Vardi's EM algorithm[1]. (See Methods for a review of Vardi's EM algorithm and how it compares with LR. See also Section 5 below for the analytical solution of LR.). This is done for the loop network (without lag, so $l = 1$) where Eq. (13) is directly applicable, and the implementation of Vardi's algorithm is reasonably doable especially since there is a unique path from each origin to each destination. Clearly, DNNGRU is superior to EM which assumes that the OD matrix comprises Poisson random variables and tries to estimate the maximum likelihood that they lead to the observed data on the edges.

The performance of LR fits between DNNGRU and EM. Intriguingly, LR is comparable to EM when T is small (i.e. given a small dataset), but asymptotically matches the performance of DNNGRU for large T. Note that DNNGRU is *always* the best, with the exception of one data point where $T = 300$ in Fig. 3(g) in the main text when predicting the most popular destination. Whilst DNNGRU can give a prediction even with $T = 1$, both LR and EM require $T \geq 11$ since the entire loop comprises 11 ADE and these methods require the observation of one full passage from O_1 to D_6 in order to calculate the necessary quantities.

Even so, DNNGRU with $T = 1$ is more accurate than LR and EM with $T = 20$, thus emphasising its superiority. Figs. 3(h, i) in the main text show the corresponding results for percentage of predictions with $\varepsilon > 0.05$ and $1 - r^2$, respectively.

5 Linear regression for a bus loop system

We consider a bus loop system where N buses pick up people from M_O origin bus stops and then let them alight at M_D destination bus stops (c.f. Fig. 1(d) in the main text). For the case of a complex network where the network is a directed loop (c.f. Fig. 4(a) in the main text), the results here for regular buses (but not semi-express buses as the network topology is different) are applicable, though we need to obtain the number of agents spawning from an origin node as well as the number of agents arriving at a destination node as the difference between two adjacent nodes.

The multivariate linear system governing the number of people boarding x_i and alighting y_j are:

$$y_j = \sum_{i=1}^{M_O} \zeta_{ij} x_i, \text{ for } j = 1, \cdots, M_D, \tag{13}$$

subject to the constraints:

$$\sum_{j=1}^{M_D} \zeta_{ij} = 1, \text{ for } i = 1, \cdots, M_O. \tag{14}$$

Note that since the M_O origins are all before the M_D destinations in the loop, the number of people alighting at the last destination is the sum of everybody who boarded minus the sum of everybody who alighted before the last destination:

$$y_{M_D} = x_1 + \cdots + x_{M_O} - y_1 - \cdots - y_{M_D - 1}. \tag{15}$$

With this and the constraints Eq. (14), we can show that the last component of Eq. (13) (where $j = M_D$) is not independent from all the other components. For instance, consider the right-hand side:

$$\begin{aligned}
\sum_{i=1}^{M_O} \zeta_{iM_D} x_i &= \sum_{i=1}^{M_O} \left(1 - \sum_{j=1}^{M_D-1} \zeta_{ij}\right) x_i \text{ using the constraints Eq. (14)} && (16)\\
&= \sum_{i=1}^{M_O} x_i - \sum_{j=1}^{M_D-1} \left(\sum_{i=1}^{M_O} \zeta_{ij} x_i\right) && (17)\\
&= \sum_{i=1}^{M_O} x_i - \sum_{j=1}^{M_D-1} y_j \text{ using Eq. (13)} && (18)\\
&= y_{M_D} \text{ using Eq. (15)}, && (19)
\end{aligned}$$

which is equal to the left-hand side. Thus, only $M_D - 1$ equations in Eq. (13) are linearly independent.

Eq. (13) is satisfied for every carrier of the agent transfer linking the nodes in a complex network, or bus going across bus stops. By linear regression, the dataset (x_i, y_j) is fitted so that the predicted $\hat{y}_j$ according to Eq. (13) as compared to the actual y_j would minimise the mean squared error:

$$J = \sum_{m,j} (\hat{y}_j - y_j)^2, \tag{20}$$

where m is the number of data points (x_i, y_j). This is a convex minimisation problem, so a global minimum of J exists, which can be analytically calculated. This involves setting $\dfrac{\partial J}{\partial \zeta_{ij}} = 0$, leading to a system of linear equations for all independent components of ζ_{ij}. We summarise the solutions for regular and semi-express bus systems with $N = M_O = M_D = 6$ in the following subsections.

5.1 Regular buses

For regular buses, every bus would board and alight commuters at every bus stop. Therefore, every bus satisfies Eq. (13). Let σ be an M_O by M_O matrix, with components:

$$\sigma_{ij} = \sum_m x_i x_j. \tag{21}$$

This leads to the following analytical solution for ζ_{ij} which minimises J. For $N = M_O = M_D = 6$, the solution is:

$$\begin{pmatrix} \zeta_{1j} \\ \zeta_{2j} \\ \zeta_{3j} \\ \zeta_{4j} \\ \zeta_{5j} \\ \zeta_{6j} \end{pmatrix} = \sigma^{-1} \cdot \begin{pmatrix} \sum_m x_1 y_j \\ \sum_m x_2 y_j \\ \sum_m x_3 y_j \\ \sum_m x_4 y_j \\ \sum_m x_5 y_j \\ \sum_m x_6 y_j, \end{pmatrix} \tag{22}$$

where $j = 1, \cdots, M_D - 1$. The $j = M_D$ components are obtained using the constraints Eq. (14).

5.2 Semi-express buses

For semi-express buses, different buses serve different subsets of origin bus stops. Therefore, different buses satisfy different versions of Eq. (13) and we need to work them out specifically. For $N = M_O = M_D = 6$, let the $N = 6$ buses be A, B, C, D, E, F. Each bus picks up from three origins, so suppose A picks up from origins $1, 2, 3$ (so $x_{A4} = x_{A5} = x_{A6} = 0$); B picks up from origins $2, 3, 4$ (so $x_{B1} = x_{B5} = x_{B6} = 0$); C picks up from origins $3, 4, 5$ (so $x_{C1} = x_{C2} = x_{C6} = 0$); D picks up from origins $4, 5, 6$ (so $x_{D1} = x_{D2} = x_{D3} = 0$); E picks up from origins $1, 5, 6$ (so $x_{E2} = x_{E3} = x_{E4} = 0$); F picks up from origins $1, 2, 6$ (so $x_{F3} = x_{F4} = x_{F5} = 0$). Then, we have the following equations for the semi-express buses A, B, C, D, E, F:

$$y_{Aj} = \zeta_{1j} x_{A1} + \zeta_{2j} x_{A2} + \zeta_{3j} x_{A3} \tag{23}$$
$$y_{Bj} = \zeta_{2j} x_{B2} + \zeta_{j3} x_{B3} + \zeta_{4j} x_{B4} \tag{24}$$
$$y_{Cj} = \zeta_{3j} x_{C3} + \zeta_{4j} x_{C4} + \zeta_{5j} x_{C5} \tag{25}$$
$$y_{Dj} = \zeta_{4j} x_{D4} + \zeta_{5j} x_{D5} + \zeta_{6j} x_{D6} \tag{26}$$
$$y_{Ej} = \zeta_{1j} x_{E1} + \zeta_{5j} x_{E5} + \zeta_{6j} x_{E6} \tag{27}$$
$$y_{Fj} = \zeta_{1j} x_{F1} + \zeta_{2j} x_{F2} + \zeta_{6j} x_{F6}, \tag{28}$$

where $j = 1, \cdots, M_D - 1$. Let $\sigma_A, \sigma_B, \sigma_C, \sigma_D, \sigma_E, \sigma_F$ be M_O by M_O matrices, with components:

$$\sigma_{Aij} = \sum_m x_{Ai} x_{Aj} \tag{29}$$
$$\sigma_{Bij} = \sum_m x_{Bi} x_{Bj} \tag{30}$$
$$\sigma_{Cij} = \sum_m x_{Ci} x_{Cj} \tag{31}$$
$$\sigma_{Dij} = \sum_m x_{Di} x_{Dj} \tag{32}$$
$$\sigma_{Eij} = \sum_m x_{Ei} x_{Ej} \tag{33}$$
$$\sigma_{Fij} = \sum_m x_{Fi} x_{Fj}, \tag{34}$$

and

$$\sigma_{\text{semi-express}} = \sigma_A + \sigma_B + \sigma_C + \sigma_D + \sigma_E + \sigma_F. \tag{35}$$

The solution is:

$$\begin{pmatrix} \zeta_{1j} \\ \zeta_{2j} \\ \zeta_{3j} \\ \zeta_{4j} \\ \zeta_{5j} \\ \zeta_{6j} \end{pmatrix} = \sigma^{-1}_{\text{semi-express}} \cdot \begin{pmatrix} \sum_m x_{A1} y_{Aj} + x_{E1} y_{Ej} + x_{F1} y_{Fj} \\ \sum_m x_{A2} y_{Aj} + x_{B2} y_{Bj} + x_{F2} y_{Fj} \\ \sum_m x_{A3} y_{Aj} + x_{B3} y_{Bj} + x_{C3} y_{Cj} \\ \sum_m x_{B4} y_{Bj} + x_{C4} y_{Cj} + x_{D4} y_{Dj} \\ \sum_m x_{C5} y_{Cj} + x_{D5} y_{Dj} + x_{E5} y_{Ej} \\ \sum_m x_{D6} y_{Dj} + x_{E6} y_{Ej} + x_{F6} y_{Fj} \end{pmatrix} \tag{36}$$

where $j = 1, \cdots, M_D - 1$. The $j = M_D$ components are obtained using the constraints Eq. (14).

6 Analytical treatment for lattice with $M_O = M_D = 3$

For this lattice, here are the shortest paths from origin nodes O_1, O_2, O_3 to D_1, D_2, D_3, respectively (c.f. Fig. 4(g) in the main text):

$$O_1 \to \begin{cases} O_2 \to D_1 \text{ or} \\ O_3 \to D_1 \text{ or} \\ D_2 \to D_1 \text{ or} \\ D_3 \to D_1, \end{cases} \tag{37}$$

$$O_1 \to D_2, \tag{38}$$

$$O_1 \to D_3, \tag{39}$$

$$O_2 \to D_1, \tag{40}$$

$$O_2 \to \begin{cases} O_3 \to D_2 \text{ or} \\ O_1 \to D_2 \text{ or} \\ D_3 \to D_2 \text{ or} \\ D_1 \to D_2, \end{cases} \tag{41}$$

$$O_2 \to D_3, \tag{42}$$

$$O_3 \to D_1, \tag{43}$$

$$O_3 \to D_2, \tag{44}$$

$$O_3 \to \begin{cases} O_1 \to D_3 \text{ or} \\ O_2 \to D_3 \text{ or} \\ D_1 \to D_3 \text{ or} \\ D_2 \to D_3. \end{cases} \tag{45}$$

Let O_1D_2 denote the number of agents on the directed edge from O_1 to D_2, and similarly for other directed edges. By inspection, we can write down this equation for the number of agents on the directed edge O_1D_2:

$$O_1D_2 = \zeta_{12}X_1 + \frac{1}{4}\zeta_{11}X_1 + O_2O_1(-1) \tag{46}$$

$$= \zeta_{12}X_1 + D_2D_1(+1) + O_2O_1(-1). \tag{47}$$

Here, X_1 is the number of agents originating from the origin node O_1 at this time step, where X_1 is not known (and is varying over time). The number of agents from O_1 going to D_2 is given by $\zeta_{12}X_1$, since ζ_{12} is the probability for an agent from O_1 going to D_2. This is the first term on the right-hand side of Eq. (46). The second term $\zeta_{11}X_1/4$ is due to the agents from O_1 having probability ζ_{11} of going to destination D_1. Amongst these agents, $1/4$ of them (on average) would take the path $O_1 \to D_2 \to D_1$, since one of the four shortest paths are selected randomly. Incidentally, this term $\zeta_{11}X_1/4$ is equal to $D_2D_1(+1)$ where "$(+1)$" implies "at the next time step" because all agents would move to D_2D_1 at the next time step. The third term $O_2O_1(-1)$ is due to agents from O_2O_1 from the previous time step now moving on to O_1D_2, where "(-1)" implies "from the previous time step".

Similarly, we obtain five more such equations:

$$O_1D_3 = \zeta_{13}X_1 + \frac{1}{4}\zeta_{11}X_1 + O_3O_1(-1) \tag{48}$$

$$= \zeta_{13}X_1 + D_3D_1(+1) + O_3O_1(-1) \tag{49}$$

$$O_2D_1 = \zeta_{21}X_2 + \frac{1}{4}\zeta_{22}X_2 + O_1O_2(-1) \tag{50}$$

$$= \zeta_{21}X_2 + D_1D_2(+1) + O_1O_2(-1) \tag{51}$$

$$O_2D_3 = \zeta_{23}X_2 + \frac{1}{4}\zeta_{22}X_2 + O_3O_2(-1) \tag{52}$$

$$= \zeta_{23}X_2 + D_3D_2(+1) + O_3O_2(-1) \tag{53}$$

$$O_3D_1 = \zeta_{31}X_3 + \frac{1}{4}\zeta_{33}X_3 + O_1O_3(-1) \quad (54)$$
$$= \zeta_{31}X_3 + D_1D_3(+1) + O_1O_3(-1) \quad (55)$$
$$O_3D_2 = \zeta_{32}X_3 + \frac{1}{4}\zeta_{33}X_3 + O_2O_3(-1) \quad (56)$$
$$= \zeta_{32}X_3 + D_2D_3(+1) + O_2O_3(-1). \quad (57)$$

Therefore, $\zeta_{12}X_1, \zeta_{13}X_1, \zeta_{21}X_2, \zeta_{23}X_2, \zeta_{31}X_3, \zeta_{32}X_3$ are given by the averages:

$$\zeta_{12}X_1 = O_1D_2 - D_2D_1(+1) - O_2O_1(-1) \quad (58)$$
$$\zeta_{13}X_1 = O_1D_3 - D_3D_1(+1) - O_3O_1(-1) \quad (59)$$
$$\zeta_{21}X_2 = O_2D_1 - D_1D_2(+1) - O_1O_2(-1) \quad (60)$$
$$\zeta_{23}X_2 = O_2D_3 - D_3D_2(+1) - O_3O_2(-1) \quad (61)$$
$$\zeta_{31}X_3 = O_3D_1 - D_1D_3(+1) - O_1O_3(-1) \quad (62)$$
$$\zeta_{32}X_3 = O_3D_2 - D_2D_3(+1) - O_2O_3(-1), \quad (63)$$

where the averages are over the measured samples of number of agents on the respective directed edges at each time step.

Incidentally by inspection, we see that for $\zeta_{11}X_1$, $\zeta_{22}X_2$ and $\zeta_{33}X_3$, these are given by the averages:

$$\zeta_{11}X_1 = D_2D_1(+1) + D_3D_1(+1) + O_1O_2 + O_1O_3 \quad (64)$$
$$\zeta_{22}X_2 = D_1D_2(+1) + D_3D_2(+1) + O_2O_3 + O_2O_1 \quad (65)$$
$$\zeta_{33}X_3 = D_1D_3(+1) + D_2D_3(+1) + O_3O_1 + O_3O_2, \quad (66)$$

where the averages are over the measured samples of number of agents on the respective directed edges at each time step. Each of the respective four terms corresponds to each of the four possible paths that can be taken.

Recall that X_1, X_2, X_3 are unknown and varying in time. Nevertheless, we can obtain all the ζ_{ij} by using the constraints Eq. (14):

$$\sum_{j=1}^{3} \zeta_{ij}X_i = X_i, \quad (67)$$

for $i = 1, 2, 3$. Therefore,

$$\zeta_{ij} = \frac{\zeta_{ij}X_i}{\sum_{k=1}^{3} \zeta_{ik}X_i}. \quad (68)$$

Note that the expression for X_i in Eq. (67) is the *average* of the samples. In fact, what we actually deduce from the edges via Eqs. (58)-(66) are these quantities $Z_{ij} = \zeta_{ij}X_i$, which allow us to get ζ_{ij} thanks to the constraint Eq. (14). Writing in terms of Z_{ij}, the probabilities ζ_{ij} are:

$$\zeta_{ij} = \frac{Z_{ij}}{\sum_{k=1}^{3} Z_{ik}}, \quad (69)$$

where we do not need to involve the unknowns X_i at all.

7 Details of the lattice, random, and small-world networks

For the lattice, random and small-world networks, respectively, as displayed in Figs. 4(b-d) of the main text, we provide the adjacency matrices, the set of shortest paths, the set of active directed edges (ADE, i.e. the edges that are actually being traversed as part of the shortest paths from O_i to D_j), as well as the set of edges used for each origin node to get to all destinations. These three networks have the same number of 12 nodes (6 origins, 6 destinations) and same number of 24 edges (or 48 directed edges). They differ in their topologies, and provide a comparison for how the resulting OD inference properties turn out. With these, the number of overlapping edges between pairs of origin nodes to get to any destinations can be computed, as presented in Table 1 of the main text.

7.1 Lattice

The adjacency matrix for the lattice A_L is:

$$A_L = \begin{pmatrix} 0&1&1&0&0&0&0&0&0&0&1&1\\ 1&0&1&1&0&0&0&0&0&0&0&1\\ 1&1&0&1&1&0&0&0&0&0&0&0\\ 0&1&1&0&1&1&0&0&0&0&0&0\\ 0&0&1&1&0&1&1&0&0&0&0&0\\ 0&0&0&1&1&0&1&1&0&0&0&0\\ 0&0&0&0&1&1&0&1&1&0&0&0\\ 0&0&0&0&0&1&1&0&1&1&0&0\\ 0&0&0&0&0&0&1&1&0&1&1&0\\ 0&0&0&0&0&0&0&1&1&0&1&1\\ 1&0&0&0&0&0&0&0&1&1&0&1\\ 1&1&0&0&0&0&0&0&0&1&1&0 \end{pmatrix}. \tag{70}$$

Here is the set of shortest paths from O_i to D_j for the lattice:

$$O_1 \to \begin{cases} O_3 \to O_5 \to D_1 \text{ or} \\ D_5 \to D_3 \to D_1, \end{cases} \tag{71}$$

$$O_1 \to \begin{cases} D_5 \to D_3 \to D_2 \text{ or} \\ D_5 \to D_4 \to D_2 \text{ or} \\ D_6 \to D_4 \to D_2, \end{cases} \tag{72}$$

$$O_1 \to D_5 \to D_3 \tag{73}$$

$$O_1 \to \begin{cases} D_5 \to D_4 \text{ or} \\ D_6 \to D_4, \end{cases} \tag{74}$$

$$O_1 \to D_5, \tag{75}$$

$$O_1 \to D_6, \tag{76}$$

$$O_2 \to \begin{cases} O_4 \to O_6 \to D_1 \text{ or} \\ O_4 \to O_5 \to D_1 \text{ or} \\ O_3 \to O_5 \to D_1, \end{cases} \tag{77}$$

$$O_2 \to \begin{cases} O_4 \to O_6 \to D_2 \text{ or} \\ D_6 \to D_4 \to D_2, \end{cases} \tag{78}$$

$$O_2 \to \begin{cases} D_6 \to D_4 \to D_3 \text{ or} \\ D_6 \to D_5 \to D_3 \text{ or} \\ O_1 \to D_5 \to D_3, \end{cases} \tag{79}$$

$$O_2 \to D_6 \to D_4, \tag{80}$$

$$O_2 \to \begin{cases} D_6 \to D_5 \text{ or} \\ O_1 \to D_5, \end{cases} \tag{81}$$

$$O_2 \to D_6, \tag{82}$$

$$O_3 \to O_5 \to D_1, \tag{83}$$

$$O_3 \to \begin{cases} O_5 \to D_1 \to D_2 \text{ or} \\ O_5 \to O_6 \to D_2 \text{ or} \\ O_4 \to O_6 \to D_2, \end{cases} \tag{84}$$

$$O_3 \to \begin{cases} O_5 \to D_1 \to D_3 \text{ or} \\ O_1 \to D_5 \to D_3, \end{cases} \tag{85}$$

$$O_3 \to \begin{cases} O_1 \to D_5 \to D_4 \text{ or} \\ O_1 \to D_6 \to D_4 \text{ or} \\ O_2 \to D_6 \to D_4, \end{cases} \tag{86}$$

$$O_3 \to O_1 \to D_5, \tag{87}$$

$$O_3 \to \begin{cases} O_1 \to D_6 \text{ or} \\ O_2 \to D_6, \end{cases} \tag{88}$$

$$O_4 \to \begin{cases} O_6 \to D_1 \text{ or} \\ O_5 \to D_1, \end{cases} \tag{89}$$

$$O_4 \to O_6 \to D_2, \tag{90}$$

$$O_4 \to \begin{cases} O_6 \to D_2 \to D_3 \text{ or} \\ O_6 \to D_1 \to D_3 \text{ or} \\ O_5 \to D_1 \to D_3, \end{cases} \tag{91}$$

$$O_4 \to \begin{cases} O_6 \to D_2 \to D_4 \text{ or} \\ O_2 \to D_6 \to D_4, \end{cases} \tag{92}$$

$$O_4 \to \begin{cases} O_2 \to D_6 \to D_5 \text{ or} \\ O_2 \to O_1 \to D_5 \text{ or} \\ O_3 \to O_1 \to D_5, \end{cases} \tag{93}$$

$$O_4 \to O_2 \to D_6, \tag{94}$$

$$O_5 \to D_1, \tag{95}$$

$$O_5 \to \begin{cases} D_1 \to D_2 \text{ or} \\ O_6 \to D_2, \end{cases} \tag{96}$$

$$O_5 \to D_1 \to D_3, \tag{97}$$

$$O_5 \to \begin{cases} D_1 \to D_3 \to D_4 \text{ or} \\ D_1 \to D_2 \to D_4 \text{ or} \\ O_6 \to D_2 \to D_4, \end{cases} \tag{98}$$

$$O_5 \to \begin{cases} O_3 \to O_1 \to D_5 \text{ or} \\ D_1 \to D_3 \to D_5, \end{cases} \tag{99}$$

$$O_5 \to \begin{cases} O_3 \to O_1 \to D_6 \text{ or} \\ O_3 \to O_2 \to D_6 \text{ or} \\ O_4 \to O_2 \to D_6, \end{cases} \tag{100}$$

$$O_6 \to D_1, \tag{101}$$

$$O_6 \to D_2, \tag{102}$$

$$O_6 \to \begin{cases} D_2 \to D_3 \text{ or} \\ D_1 \to D_3, \end{cases} \tag{103}$$

$$O_6 \to D_2 \to D_4 \tag{104}$$

$$O_6 \to \begin{cases} D_2 \to D_4 \to D_5 \text{ or} \\ D_2 \to D_3 \to D_5 \text{ or} \\ D_1 \to D_3 \to D_5, \end{cases} \tag{105}$$

$$O_6 \to \begin{cases} O_4 \to O_2 \to D_6 \text{ or} \\ D_2 \to D_4 \to D_6. \end{cases} \tag{106}$$

The set of 38 ADE for the lattice is:

$$O_1O_3, O_2O_3, O_2O_4, O_3O_4, O_3O_5, O_4O_5, O_4O_6, O_5O_6, \tag{107}$$
$$O_5D_1, O_6D_1, O_6D_2, D_1D_2, D_1D_3, D_2D_3, D_2D_4, D_3D_4, \tag{108}$$
$$D_3D_5, D_4D_5, D_4D_6, O_2O_1, O_3O_1, O_3O_2, O_4O_2, O_4O_3, \tag{109}$$
$$O_5O_3, O_5O_4, O_6O_4, D_3D_1, D_3D_2, D_4D_2, D_4D_3, D_5D_3, \tag{110}$$
$$D_5D_4, D_6D_4, D_6D_5, O_1D_5, O_1D_6, O_2D_6. \tag{111}$$

Here are the edges that are being traversed by each origin node O_i in the lattice, to get to any destination:

$$O_1 = O_1O_3, O_1D_5, O_1D_6, O_3O_5, O_5D_1, D_5D_3, D_3D_1, D_3D_2, D_5D_4, D_4D_2, D_6D_4 \tag{112}$$
$$O_2 = O_2O_4, O_2O_3, O_2D_6, O_2O_1, O_4O_6, O_6D_1, O_4O_5, O_5D_1, O_3O_5, O_6D_2, D_6D_4, D_4D_2, D_4D_3, D_6D_5, D_5D_3, O_1D_5 \tag{113}$$
$$O_3 = O_3O_5, O_3O_4, O_3O_1, O_3O_2, O_5D_1, D_1D_2, O_5O_6, O_6D_2, O_4O_6, D_1D_3, O_1D_5, D_5D_3, D_5D_4, O_1D_6, D_6D_4, O_2D_6 \tag{114}$$
$$O_4 = O_4O_6, O_4O_5, O_4O_2, O_4O_3, O_6D_1, O_5D_1, O_6D_2, D_2D_3, D_1D_3, D_2D_4, O_2D_6, D_6D_4, D_6D_5, O_2O_1, O_1D_5, O_3O_1 \tag{115}$$
$$O_5 = O_5D_1, O_5O_6, O_5O_3, O_5O_4, D_1D_2, O_6D_2, D_1D_3, D_3D_4, D_2D_4, O_3O_1, O_1D_5, D_3D_5, O_1D_6, O_3O_2, O_2D_6, O_4O_2 \tag{116}$$
$$O_6 = O_6D_1, O_6D_2, O_6O_4, D_2D_3, D_1D_3, D_2D_4, D_4D_5, D_3D_5, O_4O_2, O_2D_6, D_4D_6. \tag{117}$$

7.2 Random network

The adjacency matrix for the random network A_R is:

$$A_R = \begin{pmatrix} 0&1&0&1&0&1&1&0&1&0&0&1\\ 1&0&1&0&0&1&0&1&1&1&0&1\\ 0&1&0&0&0&0&0&1&0&0&0&0\\ 1&0&0&0&1&0&1&1&0&0&0&1\\ 0&0&0&1&0&0&0&0&1&0&1&0\\ 1&1&0&0&0&0&0&1&0&0&0&0\\ 1&0&0&1&0&0&0&0&0&0&0&0\\ 0&1&1&1&0&1&0&0&0&0&1&0\\ 1&1&0&0&1&0&0&0&0&1&0&1\\ 0&1&0&0&0&0&0&0&1&0&0&0\\ 0&0&0&0&1&0&0&1&0&0&0&1\\ 1&1&0&1&0&0&0&0&1&0&1&0 \end{pmatrix}. \tag{118}$$

Here is the set of shortest paths from O_i to D_j for the random network:

$$O_1 \to D_1, \tag{119}$$
$$O_1 \to \begin{cases} O_6 \to D_2 \text{ or} \\ O_2 \to D_2 \text{ or} \\ O_4 \to D_2, \end{cases} \tag{120}$$
$$O_1 \to D_3 \tag{121}$$
$$O_1 \to \begin{cases} O_2 \to D_4 \text{ or} \\ D_3 \to D_4, \end{cases} \tag{122}$$
$$O_1 \to D_6 \to D_5, \tag{123}$$
$$O_1 \to D_6, \tag{124}$$

$$O_2 \to O_1 \to D_1, \tag{125}$$
$$O_2 \to D_2, \tag{126}$$
$$O_2 \to D_3, \tag{127}$$
$$O_2 \to D_4, \tag{128}$$
$$O_2 \to \begin{cases} D_2 \to D_5 \text{ or} \\ D_6 \to D_5, \end{cases} \tag{129}$$
$$O_2 \to D_6, \tag{130}$$
$$O_3 \to \begin{cases} O_2 \to O_1 \to D_1 \text{ or} \\ D_2 \to O_4 \to D_1, \end{cases} \tag{131}$$
$$O_3 \to D_2, \tag{132}$$
$$O_3 \to O_2 \to D_3, \tag{133}$$
$$O_3 \to O_2 \to D_4, \tag{134}$$
$$O_3 \to D_2 \to D_5, \tag{135}$$
$$O_3 \to O_2 \to D_6, \tag{136}$$
$$O_4 \to D_1, \tag{137}$$
$$O_4 \to D_2, \tag{138}$$
$$O_4 \to \begin{cases} O_1 \to D_3 \text{ or} \\ O_5 \to D_3 \text{ or} \\ D_6 \to D_3, \end{cases} \tag{139}$$
$$O_4 \to \begin{cases} O_1 \to D_3 \to D_4 \text{ or} \\ O_5 \to D_3 \to D_4 \text{ or} \\ D_2 \to O_2 \to D_4 \text{ or} \\ D_6 \to D_3 \to D_4 \text{ (capped to 4 choices)}, \end{cases} \tag{140}$$
$$O_4 \to \begin{cases} O_5 \to D_5 \text{ or} \\ D_2 \to D_5 \text{ or} \\ D_6 \to D_5, \end{cases} \tag{141}$$
$$O_4 \to D_6, \tag{142}$$
$$O_5 \to O_4 \to D_1, \tag{143}$$
$$O_5 \to O_4 \to D_2, \tag{144}$$
$$O_5 \to D_3, \tag{145}$$
$$O_5 \to D_3 \to D_4, \tag{146}$$
$$O_5 \to D_5, \tag{147}$$
$$O_5 \to \begin{cases} O_4 \to D_6 \text{ or} \\ D_3 \to D_6 \text{ or} \\ D_5 \to D_6, \end{cases} \tag{148}$$
$$O_6 \to O_1 \to D_1, \tag{149}$$
$$O_6 \to D_2, \tag{150}$$
$$O_6 \to \begin{cases} O_1 \to D_3 \text{ or} \\ O_2 \to D_3, \end{cases} \tag{151}$$
$$O_6 \to O_2 \to D_4 \tag{152}$$
$$O_6 \to D_2 \to D_5 \tag{153}$$

$$O_6 \rightarrow \begin{cases} O_1 \rightarrow D_6 \text{ or} \\ O_2 \rightarrow D_6. \end{cases} \tag{154}$$

The set of 32 ADE for the random network is:

$$O_1O_2, O_1O_4, O_1O_6, O_1D_1, O_1D_3, O_1D_6, O_2D_2, O_2D_3, \tag{155}$$
$$O_2D_4, O_2D_6, O_3D_2, O_4O_5, O_4D_1, O_4D_2, O_4D_6, O_5D_3, \tag{156}$$
$$O_5D_5, O_6D_2, D_3D_4, D_3D_6, D_5D_6, D_2D_5, O_2O_1, O_4O_1, \tag{157}$$
$$O_6O_1, O_3O_2, O_6O_2, D_2O_2, O_5O_4, D_2O_4, D_6D_3, D_6D_5. \tag{158}$$

Here are the edges that are being traversed by each origin node O_i in the random network, to get to any destination:

$$O_1 = O_1D_1, O_1O_6, O_1O_2, O_1O_4, O_1D_3, O_1D_6, O_6D_2, O_2D_2, O_4D_2, O_2D_4, D_3D_4, D_6D_5 \tag{159}$$
$$O_2 = O_2O_1, O_2D_2, O_2D_3, O_2D_4, O_2D_6, O_1D_1, D_2D_5, D_6D_5 \tag{160}$$
$$O_3 = O_3O_2, O_3D_2, O_2O_1, O_1D_1, D_2O_4, O_4D_1, O_2D_3, O_2D_4, D_2D_5, O_2D_6 \tag{161}$$
$$O_4 = O_4D_1, O_4D_2, O_4O_1, O_4O_5, O_4D_6, O_1D_3, O_5D_3, D_6D_3, D_3D_4, D_2O_2, O_2D_4, O_5D_5, D_2D_5, D_6D_5 \tag{162}$$
$$O_5 = O_5O_4, O_5D_3, O_5D_5, O_4D_1, O_4D_2, D_3D_4, O_4D_6, D_3D_6, D_5D_6 \tag{163}$$
$$O_6 = O_6O_1, O_6D_2, O_6O_2, O_1D_1, O_1D_3, O_2D_3, O_2D_4, D_2D_5, O_1D_6, O_2D_6. \tag{164}$$

7.3 Small-world network

The adjacency matrix for the small-world network A_{SW} is:

$$A_{SW} = \begin{pmatrix} 0 & 1 & 1 & 1 & 0 & 1 & 0 & 1 & 0 & 1 & 1 & 1 \\ 1 & 0 & 1 & 1 & 0 & 0 & 0 & 0 & 0 & 0 & 0 & 0 \\ 1 & 1 & 0 & 1 & 1 & 0 & 0 & 0 & 0 & 0 & 0 & 0 \\ 1 & 1 & 1 & 0 & 1 & 0 & 0 & 1 & 0 & 0 & 0 & 0 \\ 0 & 0 & 1 & 1 & 0 & 1 & 0 & 0 & 0 & 0 & 0 & 0 \\ 1 & 0 & 0 & 0 & 1 & 0 & 1 & 0 & 0 & 0 & 0 & 0 \\ 0 & 0 & 0 & 0 & 0 & 1 & 0 & 1 & 1 & 0 & 0 & 0 \\ 1 & 0 & 0 & 1 & 0 & 0 & 1 & 0 & 1 & 1 & 1 & 0 \\ 0 & 0 & 0 & 0 & 0 & 0 & 1 & 1 & 0 & 1 & 0 & 0 \\ 1 & 0 & 0 & 0 & 0 & 0 & 0 & 1 & 1 & 0 & 1 & 0 \\ 1 & 0 & 0 & 0 & 0 & 0 & 0 & 1 & 0 & 1 & 0 & 1 \\ 1 & 0 & 0 & 0 & 0 & 0 & 0 & 0 & 0 & 0 & 1 & 0 \end{pmatrix}. \tag{165}$$

Here is the set of shortest paths from O_i to D_j for the small-world network:

$$O_1 \rightarrow \begin{cases} O_6 \rightarrow D_1 \text{ or} \\ D_2 \rightarrow D_1, \end{cases} \tag{166}$$
$$O_1 \rightarrow D_2, \tag{167}$$
$$O_1 \rightarrow \begin{cases} D_2 \rightarrow D_3 \text{ or} \\ D_4 \rightarrow D_3, \end{cases} \tag{168}$$
$$O_1 \rightarrow D_4 \tag{169}$$
$$O_1 \rightarrow D_5, \tag{170}$$
$$O_1 \rightarrow D_6, \tag{171}$$

$$O_2 \to \begin{cases} O_1 \to O_6 \to D_1 \text{ or} \\ O_1 \to D_2 \to D_1 \text{ or} \\ O_4 \to D_2 \to D_1, \end{cases} \tag{172}$$

$$O_2 \to \begin{cases} O_1 \to D_2 \text{ or} \\ O_4 \to D_2, \end{cases} \tag{173}$$

$$O_2 \to \begin{cases} O_1 \to D_2 \to D_3 \text{ or} \\ O_1 \to D_4 \to D_3 \text{ or} \\ O_4 \to D_2 \to D_3, \end{cases} \tag{174}$$

$$O_2 \to O_1 \to D_4, \tag{175}$$

$$O_2 \to O_1 \to D_5, \tag{176}$$

$$O_2 \to O_1 \to D_6, \tag{177}$$

$$O_3 \to \begin{cases} O_1 \to O_6 \to D_1 \text{ or} \\ O_1 \to D_2 \to D_1 \text{ or} \\ O_4 \to D_2 \to D_1 \text{ or} \\ O_5 \to O_6 \to D_1, \end{cases} \tag{178}$$

$$O_3 \to \begin{cases} O_1 \to D_2 \text{ or} \\ O_4 \to D_2, \end{cases} \tag{179}$$

$$O_3 \to \begin{cases} O_1 \to D_2 \to D_3 \text{ or} \\ O_1 \to D_4 \to D_3 \text{ or} \\ O_4 \to D_2 \to D_3, \end{cases} \tag{180}$$

$$O_3 \to O_1 \to D_4, \tag{181}$$

$$O_3 \to O_1 \to D_5, \tag{182}$$

$$O_3 \to O_1 \to D_6, \tag{183}$$

$$O_4 \to D_2 \to D_1, \tag{184}$$

$$O_4 \to D_2, \tag{185}$$

$$O_4 \to D_2 \to D_3, \tag{186}$$

$$O_4 \to \begin{cases} O_1 \to D_4 \text{ or} \\ D_2 \to D_4, \end{cases} \tag{187}$$

$$O_4 \to \begin{cases} O_1 \to D_5 \text{ or} \\ D_2 \to D_5, \end{cases} \tag{188}$$

$$O_4 \to O_1 \to D_6, \tag{189}$$

$$O_5 \to O_6 \to D_1, \tag{190}$$

$$O_5 \to O_4 \to D_2, \tag{191}$$

$$O_5 \to \begin{cases} O_4 \to D_2 \to D_3 \text{ or} \\ O_6 \to D_1 \to D_3, \end{cases} \tag{192}$$

$$O_5 \to \begin{cases} O_3 \to O_1 \to D_4 \text{ or} \\ O_4 \to O_1 \to D_4 \text{ or} \\ O_6 \to O_1 \to D_4 \text{ or} \\ O_4 \to D_2 \to D_4, \end{cases} \tag{193}$$

$$O_5 \rightarrow \begin{cases} O_3 \rightarrow O_1 \rightarrow D_5 \text{ or} \\ O_4 \rightarrow O_1 \rightarrow D_5 \text{ or} \\ O_6 \rightarrow O_1 \rightarrow D_5 \text{ or} \\ O_4 \rightarrow D_2 \rightarrow D_5, \end{cases} \tag{194}$$

$$O_5 \rightarrow \begin{cases} O_3 \rightarrow O_1 \rightarrow D_6 \text{ or} \\ O_4 \rightarrow O_1 \rightarrow D_6 \text{ or} \\ O_6 \rightarrow O_1 \rightarrow D_6, \end{cases} \tag{195}$$

$$O_6 \rightarrow D_1, \tag{196}$$

$$O_6 \rightarrow \begin{cases} O_1 \rightarrow D_2 \text{ or} \\ D_1 \rightarrow D_2, \end{cases} \tag{197}$$

$$O_6 \rightarrow D_1 \rightarrow D_3 \tag{198}$$

$$O_6 \rightarrow O_1 \rightarrow D_4 \tag{199}$$

$$O_6 \rightarrow O_1 \rightarrow D_5 \tag{200}$$

$$O_6 \rightarrow O_1 \rightarrow D_6. \tag{201}$$

The set of 24 ADE for the small-world network is:

$$O_1O_6, O_1D_2, O_1D_4, O_1D_5, O_1D_6, O_2O_4, O_2O_1, O_3O_4, \tag{202}$$

$$O_3O_5, O_3O_1, O_4D_2, O_4O_1, O_5O_6, O_5O_3, O_5O_4, O_6D_1, \tag{203}$$

$$O_6O_1, D_1D_2, D_1D_3, D_2D_3, D_2D_4, D_2D_5, D_2D_1, D_4D_3. \tag{204}$$

Here are the edges that are being traversed by each origin node O_i in the small-world network, to get to any destination:

$$O_1 = O_1O_6, O_1D_2, O_1D_4, O_1D_5, O_1D_6, O_6D_1, D_2D_1, D_2D_3, D_4D_3 \tag{205}$$

$$O_2 = O_2O_1, O_2O_4, O_1O_6, O_6D_1, O_1D_2, D_2D_1, O_4D_2, D_2D_3, O_1D_4, D_4D_3, O_1D_5, O_1D_6 \tag{206}$$

$$O_3 = O_3O_1, O_3O_4, O_3O_5, O_1O_6, O_6D_1, O_1D_2, D_2D_1, O_4D_2, O_5O_6, D_2D_3, O_1D_4, D_4D_3, O_1D_5, O_1D_6 \tag{207}$$

$$O_4 = O_4D_2, O_4O_1, D_2D_1, D_2D_3, O_1D_4, D_2D_4, O_1D_5, D_2D_5, O_1D_6 \tag{208}$$

$$O_5 = O_5O_6, O_5O_4, O_5O_3, O_6D_1, O_4D_2, D_2D_3, D_1D_3, O_3O_1, O_1D_4, O_4O_1, O_6O_1, D_2D_4, O_1D_5, D_2D_5, O_1D_6 \tag{209}$$

$$O_6 = O_6D_1, O_6O_1, O_1D_2, D_1D_2, D_1D_3, O_1D_4, O_1D_5, O_1D_6. \tag{210}$$

References

1. Vardi, Y. Network tomography: Estimating source-destination traffic intensities from link data. *J. Am. Stat. Assoc.* **91**, 365–377, DOI: 10.1080/01621459.1996.10476697 (1996).

2. Tebaldi, C. & West, M. Bayesian inference on network traffic using link count data. *J. Am. Stat. Assoc.* **93**, 557–573, DOI: 10.1080/01621459.1998.10473707 (1998).

3. Van Zuylen, J. H. & Willumsen, L. G. The most likely trip matrix estimated from traffic counts. *Transp. Res. Part B: Methodol.* **14**, 281–293, DOI: https://doi.org/10.1016/0191-2615(80)90008-9 (1980).

4. Dey, S. & Fricker, J. Bayesian updating of trip generation data: Combining national trip generation rates with local data. *Transportation* **21**, 393 (1994).

5. Carvalho, L. A bayesian statistical approach for inference on static origin–destination matrices in transportation studies. *Technometrics* **56**, 225–237, DOI: 10.1080/00401706.2013.826144 (2014). https://doi.org/10.1080/00401706.2013.826144.

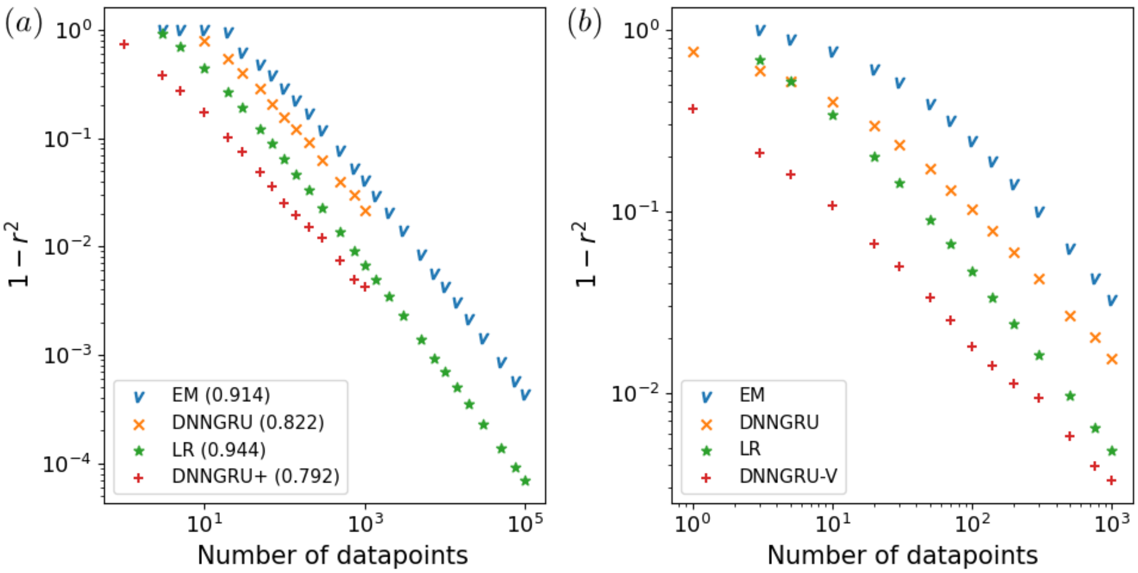

Figure S1. a: The same graph as Fig. 3(c) in the main text, with more datapoints for EM and LR up till 10^5. Shown in the parentheses of the legend are the corresponding exponents of the power law fits. The results for DNNGRU and DNNGRU-V are not produced beyond 1000 datapoints because they become computationally infeasible to train. Nevertheless, it is apparent by extrapolation that DNNGRU-V would converge to LR's performance (or even begin to become inferior) in the asymptotic limit with large datapoints. b: The corresponding results with respect to Fig. 3(c) in the main text, where the various methods are to predict the actual OD instead of ζ_{ij}. Just like predicting ζ_{ij}, LR, DNNGRU-V and DNNGRU are superior to Vardi's expectation-maximisation in predicting the actual OD.

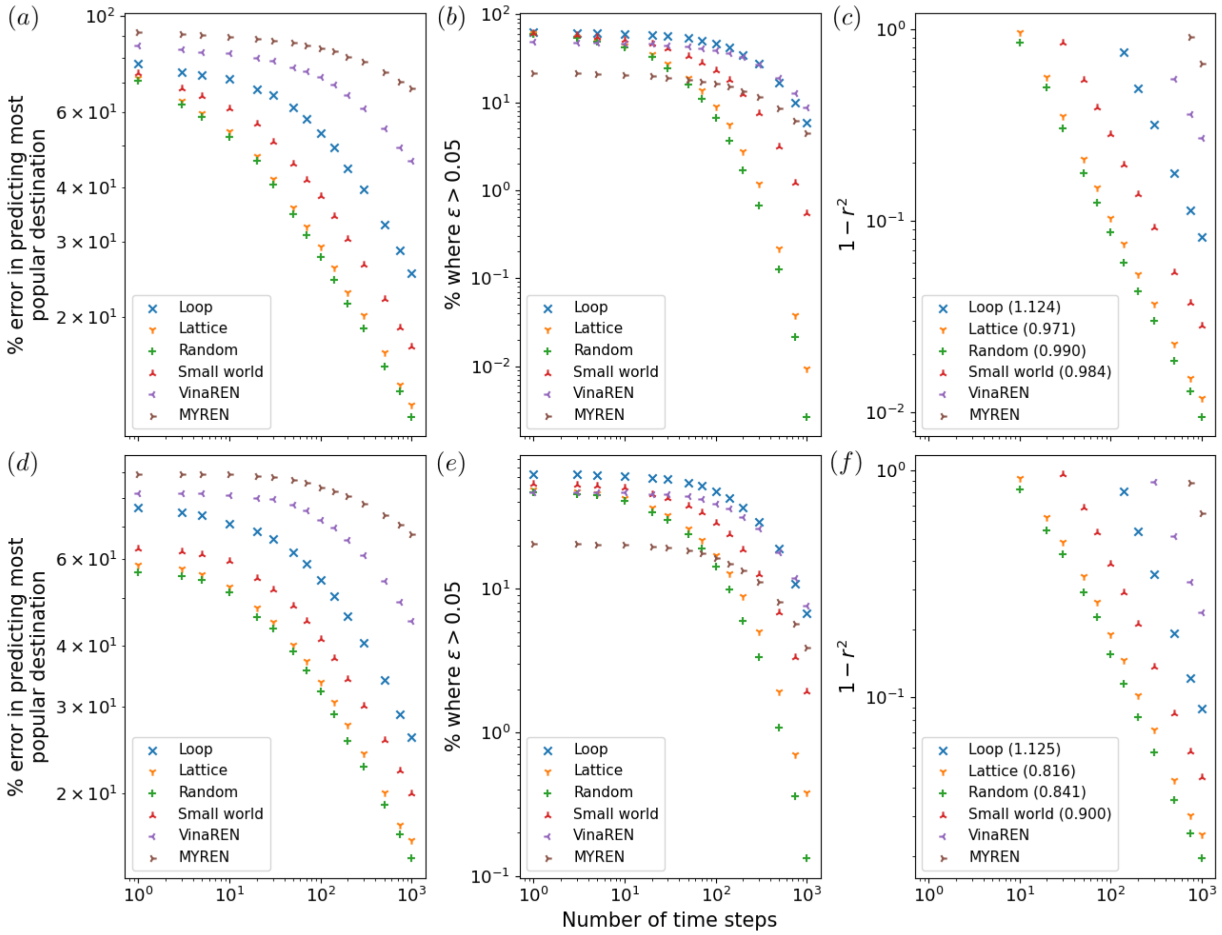

Figure S2. The corresponding results with respect to Fig. 6 in the main text. The top row is for no lag ($l = 1$) traversing the edges, whilst the bottom row is with lag of $l = 10$ time steps.

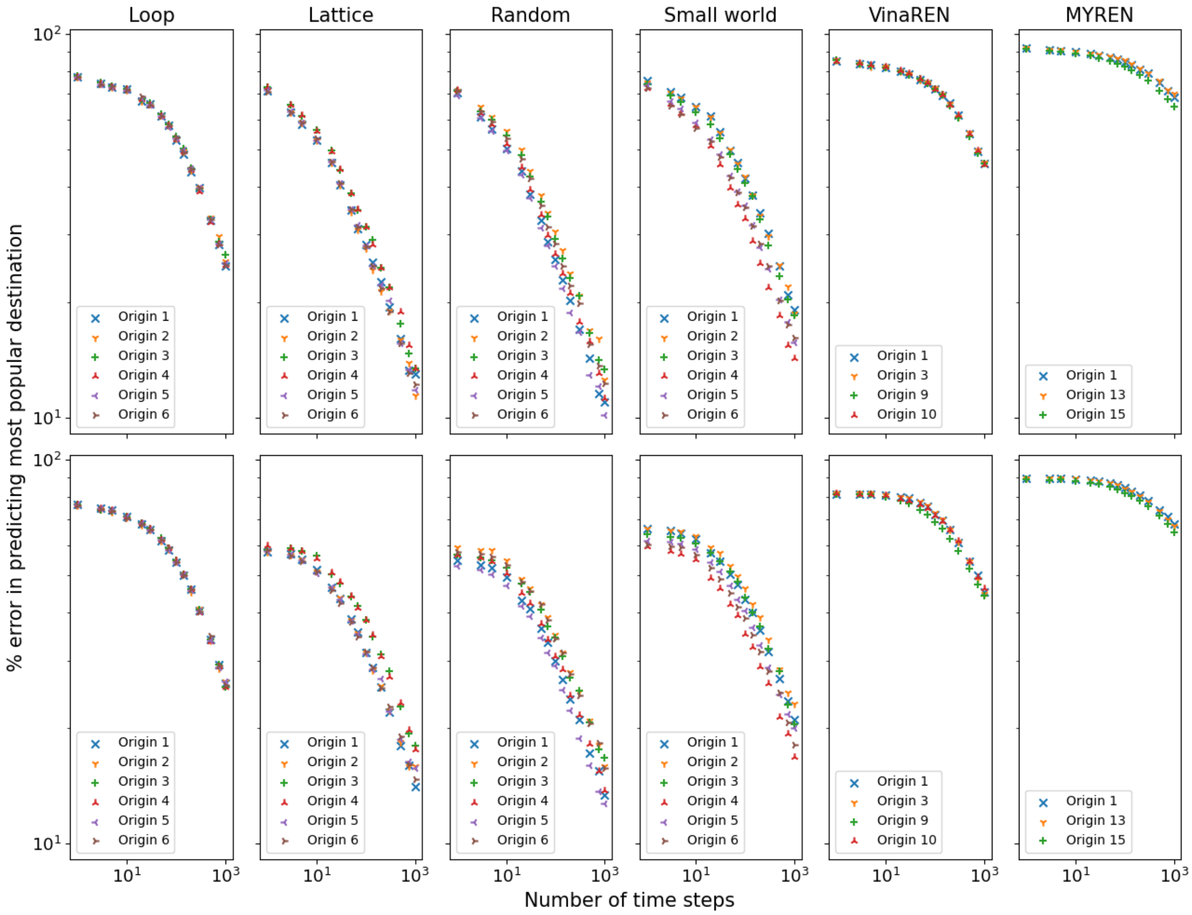

Figure S3. The corresponding results with respect to Fig. 7 in the main text, for percentage error in predicting the most popular destination. The top row is for no lag ($l = 1$) traversing the edges, whilst the bottom row is with lag of $l = 10$ time steps.

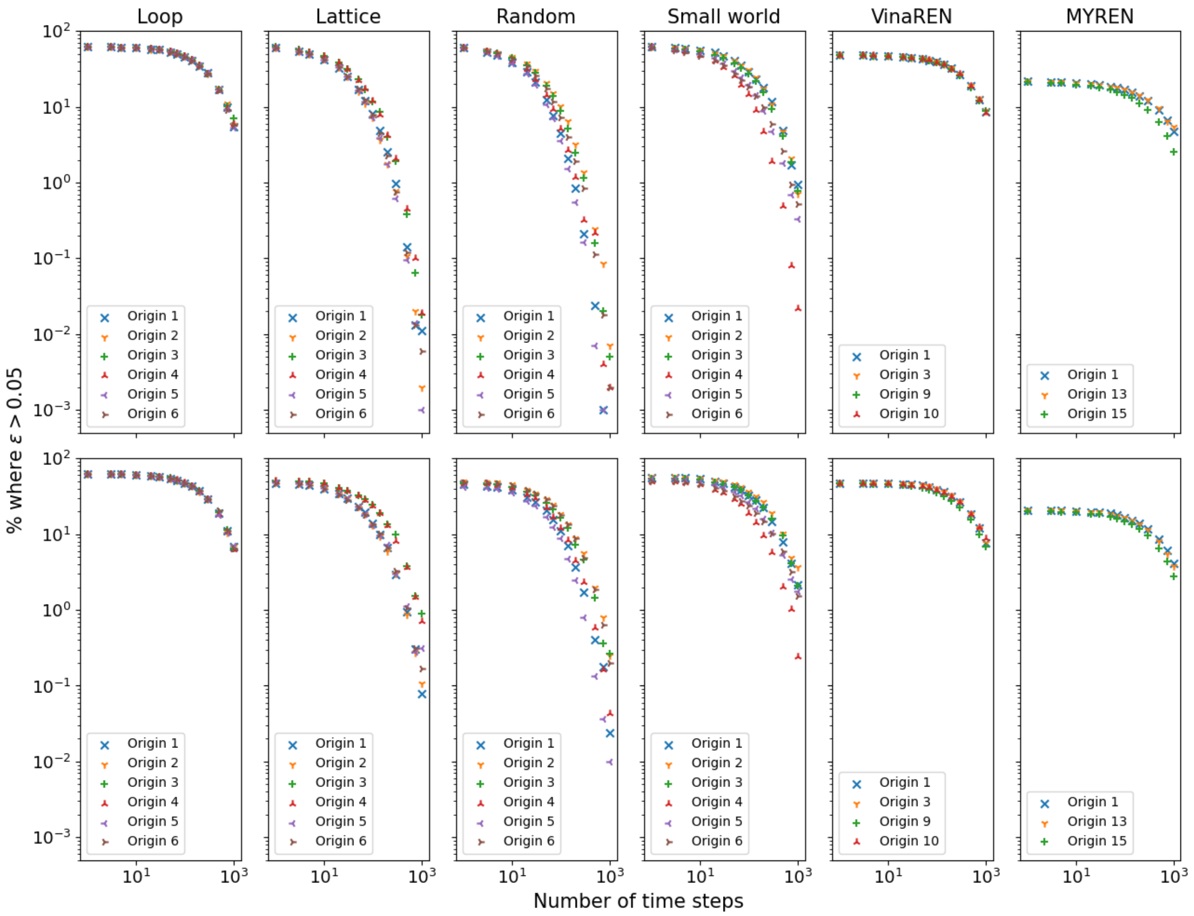

Figure S4. The corresponding results with respect to Fig. 7 in the main text, for percentage of predictions where $\varepsilon > 0.05$. The top row is for no lag ($l = 1$) traversing the edges, whilst the bottom row is with lag of $l = 10$ time steps.

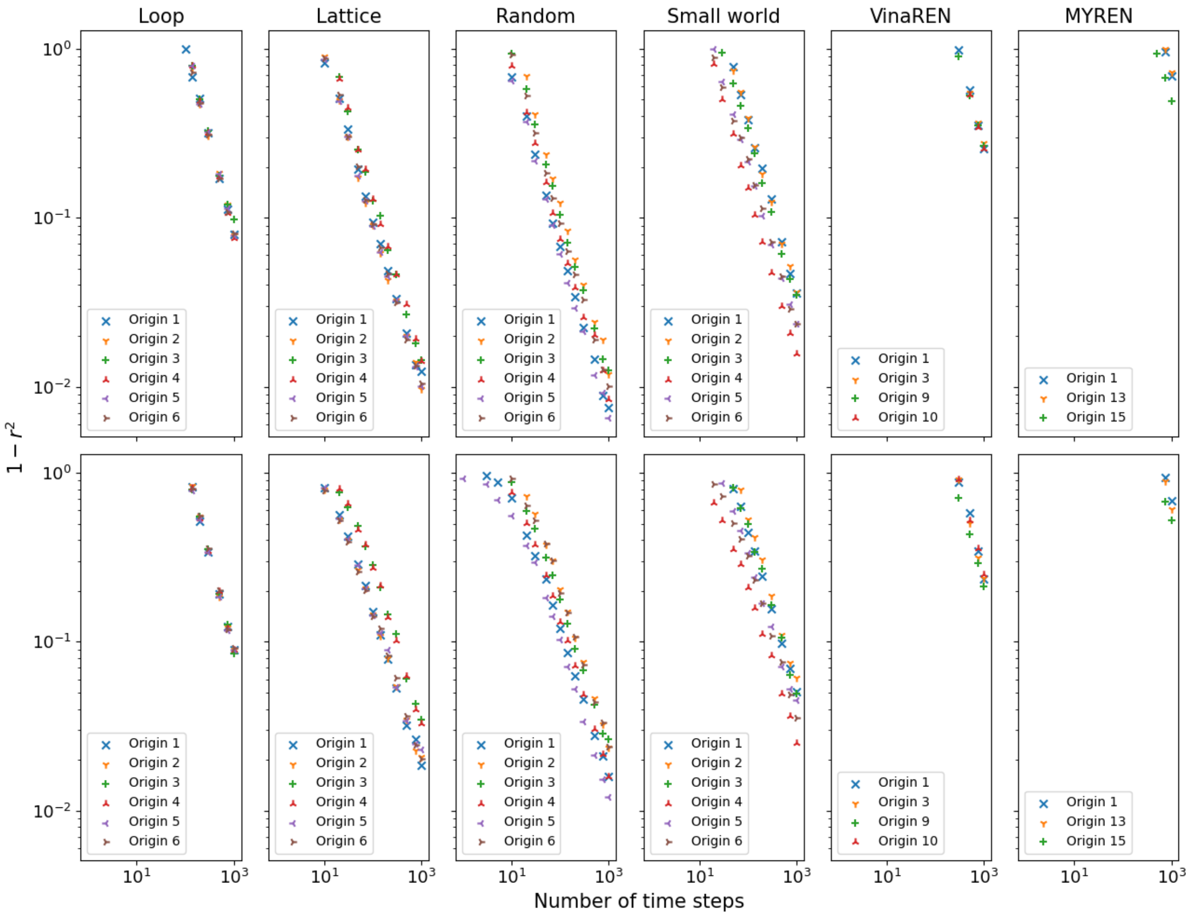

Figure S5. The corresponding results with respect to Fig. 7 in the main text, for $1-r^2$. The top row is for no lag ($l=1$) traversing the edges, whilst the bottom row is with lag of $l=10$ time steps.
